# Metabolic dysfunction and mitochondrial failure in Alzheimer's disease: integrating pathophysiology, clinical evidence and emerging interventions

**DOI:** 10.3389/fneur.2026.1772036

**Published:** 2026-02-26

**Authors:** Xiaohua Xiao, Xueqin Yan, Chunhua Liang, Yunzhu Yang

**Affiliations:** Geriatrics Department, Shenzhen Second People's Hospital, Guangdong, China

**Keywords:** Alzheimer's disease, brain energy metabolism, emerging interventions, insulin resistance, metabolic dysfunction, mitochondrial failure, neurodegeneration, oxidative stress

## Abstract

Alzheimer's disease (AD) is a gradual and irreversible decline in the brain's ability to function which is not only signified by amyloid-beta plaques and neurofibrillary tangles but also by and metabolic and mitochondrial changes that have a negative impact on the classical neuropathological hallmarks. It is becoming increasingly clear that the central roles in the process of synaptic dysfunction, neuronal death and cognitive decline are played by the brain's impaired glucose utilization, insulin resistance, lipid metabolism alterations, and energy homeostasis disruption. Mitochondrial dysfunctions in AD comprising of oxidative phosphorylation defects, ATP production decrease, reactive oxygen species generation over and above the normal level, poor mitochondrial dynamics, and vacuolar-type H+-ATPase-mediated cell death are the factors that further worsen the situation and hence speed up the process of neuronal death and eventually, disease progression. The metabolic and mitochondrial disturbances have a two-way relationship with amyloid-beta and tau pathology, neuroinflammation, and oxidative stress, thus creating a self-sustaining cycle of neurodegeneration. Besides, clinical and neuroimaging studies, fluorodeoxyglucose positron emission tomography, cerebrospinal fluid biomarkers, and peripheral metabolic profiling all support the notion that metabolic impairment is an early and clinically relevant feature of AD very convincingly. Thus, the attention of the scientific community has turned more and more toward the approaches that use the metabolic and mitochondrial pathways as their target. The new treatments are coming, including insulin sensitizers, ketogenic and Mediterranean diets, mitochondrial-targeted antioxidants, exercise, metabolic modulators, and new drugs, all aimed at bringing back equilibrium to bioenergetics and letting neurons live longer. In this review, we have considered the current mechanistic insights, clinical evidence, and therapeutic advances related to metabolic dysfunction and mitochondrial failure in AD together and their potential as early biomarkers and modifiable targets for disease prevention and treatment that are highlighted.

## 1 Introduction

Alzheimer's disease (AD) remains leading cause of cognitive impairment and dementia in older persons (aged ≥ 65 years) throughout the world (1). The prevalence now impacts 3%−4% of the late-working population, with statistics in China ranging from 0.2% among individuals aged 55 to over 48% in those aged 95 and older (2–4). It is anticipated that, by 2050, there will be 152 million individuals with Alzheimer's disease along with other dementias (5). The Lancet Commission has identified numerous modifiable risk factors, including hypertension, diabetes, physical inactivity, and air pollution, that significantly contribute to the disease burden, in addition to aging (6).

The conventional amyloid-centric perspective of Alzheimer's disease emphasizes the metabolic dysfunction of amyloid precursor protein (APP) and the resultant aggregation of amyloid-beta (Aβ). Aβ amyloidosis induces neuroinflammation and affects Tau pathology—a microtubule-associated protein crucial for tubulin assembly—yet recent research indicates that these may represent separate clinical processes (7). Tau pathology is significantly correlated with the advancement of Alzheimer's disease and associated risk factors such as ApoE4 and TREM2 (8, 9).

Current evidence indicates that cellular impacts occur years prior to the onset of clinical symptoms, with mitochondrial failure in neurones and astrocytes acting as a primary early indicator (10, 11). The dysfunction of this organelle frequently occurs prior to the characteristic aggregation of proteins, establishing metabolic health as a pivotal factor in the disease (12). Consequently, mitochondria-targeted interventions, such as antioxidants, have shown promise in slowing disease evolution (13).

While Aβ and Tau have been the main subjects of prior study, new data emphasizes mitochondrial breakdown and the ensuing oxidative stress as early, predictable events in AD pathogenesis. This review sets itself apart by putting up an Integrative Metabolic-Biomarker Framework that connects certain clinical imaging and fluid biomarkers to early bioenergetic abnormalities. In order to give a strategic roadmap for mitochondria-based interventions in the era of precision medicine, we offer a critical evaluation of the “translational gap” in metabolic medicines, such as GLP-1 RAs and mitophagy inducers, beyond a comprehensive overview of energy pathways.

## 2 Brain energy metabolism in health

### 2.1 Overview of neuronal glucose utilization

The brain is an energy-intensive organ requiring a constant supply of ATP to maintain the amount of ions across the neuronal membrane, depolarising neurones and support neurotransmission. Because the brain lacks large triglyceride storage and retains only small glycogen reserves, it relies on a constant input of blood-borne metabolites (14). The blood-brain barrier (BBB), a highly selective semipermeable membrane that permits the passive diffusion of small hydrophobic molecules like O_2, CO_2, and ethanol while shielding the neuronal environment from infections and big hydrophilic molecules, controls this influx (15).

Glucose is the brain's principal fuel and a necessary precursor for the synthesis of lipids, proteins, and neurotransmitters like acetylcholine, glutamate, and gamma-aminobutyric acid (GABA). Specific sodium-independent glucose transporters (GLUTs) facilitate transport across the BBB: GLUT1 is largely expressed in endothelial cells and astrocytes, whereas GLUT3 and GLUT4 are localized to neurones (16). Once inside astrocytes and neurones, glucose undergoes glycolysis, converting to pyruvate and producing ATP.

While glucose is the primary metabolic fuel, the brain may actively transport and use alternate substrates through specialized carrier proteins. These include glutamate, pyruvate, lactate, and ketones (17). This metabolic flexibility is critical during times of glucose scarcity or high functional demand.

### 2.2 Mitochondrial oxidative phosphorylation

To satisfy bioenergetic needs, cellular metabolism combines the breakdown of proteins, fats, and carbohydrates. These processes come together to form the tricarboxylic acid (TCA) cycle through glycolysis, fatty acid beta-oxidation, and amino acid transamination (18). Located within the mitochondrial matrix, the TCA cycle produces the reduced cofactors NADH and FADH_2, which serve as the primary electron donors for the electron transport chain (ETC).

The inner mitochondrial membrane (IMM) incorporates five protein complexes that collectively make up the ETC. Complex I (NADH: ubiquinone oxidoreductase) or Complex II (succinate dehydrogenase) carry electrons to the ETC. These electrons are then shuttled to ubiquinone (Q), reducing it to ubiquinol (QH_2). Four protons (H+) are injected into the intermembrane space (IMS) as electrons transit from Complex I to the Q cycle. Interestingly, Complex II has no role in the translocation of protons.

Complex III then oxidizes ubiquinol, facilitating the transport of electrons to cytochrome c. Four more protons are introduced into the IMS for each electron pair passing through this cycle (19). This sequential transfer establishes the electrochemical gradient necessary for ATP synthesis.

### 2.3 Astrocyte-neuron lactate shuttle

The astrocyte-neuron lactate shuttle (ANLS) hypothesis, the foundation of cerebral energy balance is a basic metabolic cooperation between astrocytes and neurones (20, 21). According to this framework, astrocytes serve as the primary site for glucose uptake and its subsequent conversion into lactate. This lactate is then tansported to neurons, where it is oxidized back into pyruvate to fuel the tricarboxylic acid (TCA) cycle for ATP production. It is a pathway that may offer higher energetic efficiency than direct glucose utilization.

Beyond its role as an energy substrate, lactate acts as a crucial signaling molecule in the brain. Recent studies have highlighted its numerous physiological functions, including the modulation of synaptic plasticity, memory consolidation, and neuroprotection (22). Consequently, disruptions in lactate homeostasis are linked to a number of neurological conditions, including as Alzheimer's disease (23), traumatic brain injury (24), and epilepsy (25).

### 2.4 Regulation of redox homeostasis and ATP production

The central nervous system (CNS) is the primary coordinator of physiological function, yet its high metabolic demand renders it uniquely vulnerable. Despite accounting for a small fraction of body mass, the brain consumes approximately 20% of the body's total glucose and oxygen—a rate ten times higher than most other tissues (26). This intense metabolic activity significantly increases the production of free radicals, especially reactive oxygen species (ROS). The brain uses energy from several sources, primarily glucose, to maintain the redox balance in the neuronal and glial cells in order to defend itself against oxidative damage by having an adequate antioxidant system (27).

ROS are continuously generated within brain cells via the mitochondrial electron transport chain and various enzymatic pathways under both physiological and pathological conditions. For instance, as part of normal signaling, monoamine oxidases (MAO-A and MAO-B) metabolize catecholamines, yielding hydrogen peroxide as a byproduct. Furthermore, NADPH oxidases can be pathologically activated by ischemia, seizures, protein misfolding, and overactivity of the glutamatergic system (28, 29).

The metabolic rate and signaling intensity can vary because different regions of the brain are specialized for distinct functions. This heterogeneity extends to the distribution of endogenous antioxidants, such as glutathione (GSH) (30, 31). Consequently, there may be variations in GSH levels between anatomical regions as well as between cell types. Crucially, physical exercise and toxic exposures can produce significant changes in the levels of antioxidants, particularly GSH, in various regions of the brain (32).

## 3 Mitochondrial dysfunction in alzheimer's disease

### 3.1 Structural alteration in mitochondria

Mitochondria are central to the neuropathology of Alzheimer's disease (AD), regulating oxidative stress, intracellular Ca2+ signaling, and glutamate-mediated synaptic transmission (33). Disruptions in mitochondrial function trigger Ca2+ imbalances that drive synaptotoxicity and subsequent cognitive decline (34). Furthermore, impaired mitophagy and other mitochondrial quality control mechanisms are critical to the pathophysiology of AD (35).

In AD, the structural integrity and motility of mitochondria are severely altered. Evidence suggests that neuronal mitochondria in the AD brain exhibit fewer cristae and a condensed, darker matrix, characterized by increased fission and diminished fusion (36). These dysregulated dynamics—a common hallmark across neurodegenerative disorders, including Alzheimer‘s, Parkinson's, and Huntington's diseases, lead to mitochondrial fragmentation, reduced bioenergetic efficiency, and elevated oxidative stress (37).

**Figure 1:**
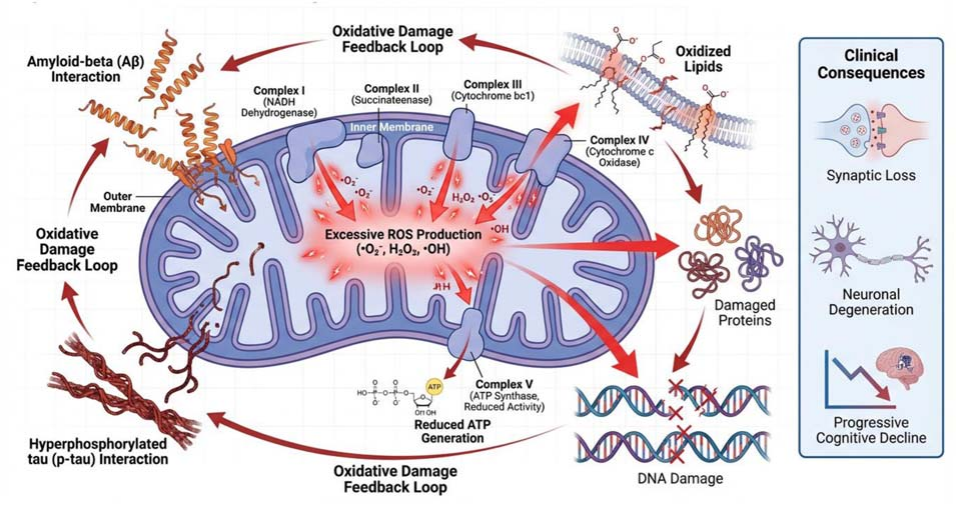
Mitochondrial bioenergetic failure and oxidative stress in Alzheimer's disease.

The association of beta-Amyloid with mitochondrial components induces profound ultrastructural changes and oxidative damage, often mediated by disruptions in AMP-activated protein kinase (AMPK) pathways (38, 39). These strictly regulated processes of fusion and fission, collectively termed mitochondrial dynamics, are essential for maintaining optimal organelle distribution and cellular function (40). When these mechanisms fail, the resulting energy deficits and neuronal injury accelerate the progression of AD (41) (Figure 1).

Transgenic AD mouse models, including 5xFAD, FAD, and APP, have been shown to exhibit mitophagy, Aβ buildup in synaptosomal mitochondria, disruptions in mitochondrial dynamics indicators, and structural alterations (42). Studies focusing on the dysregulation of important mitochondrial fission and fusion proteins, including OPA1, MFN2, and DRP1, have demonstrated notable disturbances in mitochondrial dynamics that may aid in the advancement of disease. However, more research is needed to fully understand the time-dependent evolution of the alterations in mitochondrial dynamics markers in transgenic AD mice that exhibit classic AD pathologies, such as amyloid plaques and cellular degeneration. Furthermore, although AD brain tissue frequently exhibits mitochondrial failure, new research has shown that peripheral mononuclear blood cells' mitochondrial activities have changed (11).

### 3.2 Impaired electron transport chain function

Alzheimer's disease is marked by decreased activity in several enzymatic complexes critical for ATP production, including the pyruvate dehydrogenase complex, α-ketoglutarate dehydrogenase complex, complex I (NADH ubiquinone oxidoreductase), complex IV (cytochrome oxidase), and complex V (ATPase) (43, 44). Additionally, the activities of key glycolytic enzymes—phosphofructokinase (PFK), phosphoglycerate mutase, aldolase, glucose-6-phosphate isomerase, and lactate dehydrogenase—are reduced compared to age-matched non-Alzheimer's brains. Conversely, both the levels and activity of reactive oxygen species (ROS) are elevated. Moreover, mitochondrial translocase facilitates the progressive accumulation of mitochondrial Aβ in the brains of transgenic mice overexpressing human APP, which correlates with diminished activity of electron transport chain complexes III and IV and a decreased rate of oxygen consumption (43).

### 3.3 Defective mitochondrial biogenesis

Maintaining an adequate pool of functional neural mitochondria and restoring those that have been damaged or lost relies on mitochondrial biogenesis. This tightly regulated process requires coordination between the nuclear and mitochondrial genomes. Mitochondrial biogenesis occurs continuously in healthy cells, as mitochondria undergo constant proliferation and fusion, but it can also be induced by oxidative stress, increased energy demands, physical activity, and certain diseases (45). However, the status of mitochondrial biogenesis in neurons affected by Alzheimer's disease (AD) remains unclear. Peroxisome proliferator-activated receptor gamma coactivator-1 alpha (PGC-1α) governs this process by activating multiple transcription factors, including nuclear respiratory factors 1 and 2 (NRF-1 and NRF-2) and mitochondrial transcription factor A (TFAM) (46). NRF-1 and NRF-2 regulate nuclear and mitochondrial genes essential for various critical functions, such as oxidative phosphorylation (OXPHOS), electron transport chain complexes I–V, mitochondrial DNA (mtDNA) transcription and replication, protein import and assembly, ion channel activity, molecular transport, and translation (47). Additionally, in response to oxidative stress, PGC-1α orchestrates a complex reactive oxygen species (ROS) defense system vital for maintaining redox homeostasis. This highlights the crucial role of mitochondrial biogenesis in preserving mitochondrial integrity throughout the organelle's lifespan. Notably, key components of the electron transport chain (ETC) are significantly diminished in AD, contributing to energy hypometabolism and mitochondrial dysfunction.ay indicate either increased mitochondrial clearance or decreased mitochondrial biogenesis (48).

### 3.4 Calcium dysregulation and mitochondrial permeability transition pore opening

Mitochondria are very important for maintaining stable calcium levels in the cytosol because they facilitate calcium entry and exit through several different pathways. This uptake is most efficient at high-Ca2+ microdomains, particularly within Mitochondria-Associated Membranes (MAMs)—specialized sub-cellular regions where the endoplasmic reticulum (ER) and mitochondria exist in close apposition (49). Various illnesses, such as cancer, neurological conditions, and metabolic syndromes, have been linked to changes in MAM structure or function.

One of the most common and early indicator of brain disorders, such as AD, is altered cellular calcium homeostasis (50). Altered Ca2+ homeostasis is a recognized early hallmark of AD (51):

1. excessive calcium influx disrupts homeostasis, resulting in neuronal injury and dysfunction.
2. elevated intracellular calcium promotes Aβ accumulation.
3. calcium overload leads to abnormal tau phosphorylation, resulting in NFT formation.
4. calcium imbalance impairs synaptic plasticity, which contributes to cognitive decline.

Extracellular oligomeric Aβ further exacerbates this instability by activating membrane receptors and forming *de novo* Ca2+-permeable pores in the plasma membrane (52). According to the current paradigm, glutamate-driven excitotoxicity, activation of calcium-dependent proteases, over activation of calcium-regulated kinases and phosphatases, and mitochondrial calcium overload resulting in mPTP opening (53, 54) are some of the mechanisms by which generalized calcium overload in neurons causes neuronal death (54, 55).

### 3.5 Mitochondrial DNA mutations and oxidative damage

Each mitochondrion contains multiple copies of mitochondrial DNA (mtDNA), which is particularly susceptible to oxidative damage and somatic mutations due to its close proximity to the electron transport chain (56). The progressive accumulation of mtDNA mutations, along with increased reactive oxygen species (ROS) and disrupted mitochondrial dynamics, severely compromises mitochondrial membrane potential and exacerbates organelle dysfunction. Loss of mitochondrial integrity is critical in this process. Under severe oxidative stress, oligomerization of voltage-dependent anion channel 1 (VDAC1) facilitates the formation of large pores in the outer mitochondrial membrane. These pores allow mtDNA to move from the matrix into the cytosol, a key event that triggers inflammatory signaling and further cellular deterioration (57) (Table 1).

**Table 1:** Biomarkers linking metabolic and mitochondrial dysfunction to Alzheimer's disease.

**Biomarker**	**Biological source**	**Pathophysiological link**	**Diagnostic/prognostic value**	**References**
FDG uptake	Brain tissue	Glucose metabolism	Early detection	(271)
CSF lactate	CSF	Mitochondrial stress	Disease severity	(272)
mtDNA copy number	Blood/CSF	Mitochondrial health	Progression marker	(273)
Oxidized lipids	Plasma	Oxidative stress	Risk assessment	(274)
NAD^+^ levels	CSF	Redox imbalance	Therapeutic target	(11)
ATP concentration	Neurons	Energy status	Functional decline	(275)
Insulin receptor expression	Brain tissue	Insulin resistance	Pathology link	(276)
Drp1 levels	Neurons	Excessive fission	Synaptic damage	(277)
PGC-1α expression	Brain	Biogenesis capacity	Neuroprotection	(278)
ROS markers	Plasma	Oxidative burden	Disease activity	(279)
SOD2 activity	Mitochondria	Antioxidant defense	Protective indicator	(280)
Tau phosphorylation	CSF	Mitochondrial interaction	Disease stage	(44)
Amyloid-β oligomers	CSF	Metabolic toxicity	Early pathology	(281)
Lactate shuttle enzymes	Astrocytes	Energy coupling	Network stability	(282)
Inflammatory cytokines	Plasma	Metabolic inflammation	Progression risk	(283)
Mitochondrial membrane potential	Neurons	Bioenergetic integrity	Cell survival	(284)
Ketone utilization markers	Brain	Fuel flexibility	Intervention response	(285)

### 3.6 Mitophagy impairment:PINK1 parkin and alternative pathways

Mitophagy is a specialized form of autophagy essential for mitochondrial quality control, involving the selective sequestration of damaged mitochondria into autophagosomes for lysosomal degradation. Impaired mitophagy is a hallmark of biological aging and is closely linked to the pathogenesis of neurodegenerative disorders, most notably Alzheimer's and Parkinson's diseases (33). The canonical mitophagy pathway is triggered by depolarization of the inner mitochondrial membrane. Under physiological conditions, the kinase PINK1 is rapidly degraded; however, when mitochondrial membrane potential is lost, PINK1 stabilizes on the outer mitochondrial membrane (OMM). Once stabilized, PINK1 phosphorylates Mitofusin 2 (Mfn2) and ubiquitin, facilitating the recruitment of the E3 ubiquitin ligase Parkin to the OMM (58). The recruitment of Parkin activates the ubiquitin-proteasome system (UPS) and signals the phagophore to engulf the compromised organelle. This process culminates in the formation of a mitophagosome, which then fuses with a lysosome for enzymatic degradation. The pivotal roles of the PINK1-Parkin axis in maintaining mitochondrial integrity have been extensively documented in various experimental models (58).

## 4 Metabolic dysfunction in alzheimer's disease

### 4.1 Cerebral glucose hypometabolism: FDG-PET evidence

Fluorodeoxyglucose positron emission tomography (FDG-PET) remains a cornerstone for assessing cerebral glucose metabolism, providing a reliable proxy for synaptic density and neuronal energy demand. Unlike biomarkers that reflect downstream pathological accumulation, glucose hypometabolism can identify early functional deficits within neural networks. These metabolic changes often appear years before irreversible structural atrophy occurs, making them invaluable for early detection (59, 60). Emerging research indicates that metabolic dysfunction is not just a symptom but a critical determinant of an individual's disease trajectory and cognitive decline (61). Clarifying how these metabolic changes affect different progression pathways could fundamentally shift the Alzheimer's disease paradigm from a reactive diagnostic model to a proactive, pathway-guided management strategy. This transition would support the development of precision medicine interventions tailored to the unique physiological trajectory of each patient (62).

### 4.2 Brain insulin resistance (Type 3 diabates)

A controversial but intriguing concept for understanding Alzheimer's disease (AD) is the “Type 3 Diabetes” (T3D) hypothesis, which suggests that AD may be a form of brain-specific insulin resistance. According to this theory, when neurons become insensitive to insulin, crucial cognitive functions like memory consolidation and synaptic plasticity are significantly impaired. A key player in this hypothesis is the insulin-degrading enzyme (IDE). In conditions of high insulin levels, IDE may be overwhelmed by peripheral insulin, reducing its ability to break down amyloid-beta (Aβ) and leading to the accumulation of toxic proteins (63). While insulin resistance is a shared feature among obesity, Type 2 Diabetes (T2D), and AD, the manifestation in the central nervous system (CNS) is unique. In the brain, resistance is mainly characterized by disrupted signaling pathways, such as the PI3K/Akt pathway, rather than a primary issue with glucose transport, as neuronal glucose uptake is largely independent of insulin.

However, it is important to acknowledge that the terminology for T3D is still a topic of discussion. The relationship between T2D and cognitive decline in AD is well-established, but the evidence is varied. AD pathology is now seen as a complex network where insulin signaling issues, Aβ accumulation, and tau hyperphosphorylation interact and worsen each other within the A/T/N framework (Amyloid/Tau/Neurodegeneration) (64).

### 4.3 Impaired glycolytic flux and TCA cycle disruption

Extensive research has highlighted the association between AD and the dysregulation of key glycolytic enzymes, including hexokinase, glyceraldehyde 3-phosphate dehydrogenase (GAPDH), and pyruvate kinase (PK) (65, 66). The pathogenesis of AD is closely linked to the tricarboxylic acid (TCA) cycle; specifically, the accumulation of Aβ and the hyperphosphorylation of Tau inhibit TCA cycle activity. This inhibition leads to critical ATP depletion, which accelerates neuronal death (65, 66). The metabolic decline is further worsened by the accumulation of reactive oxygen species (ROS), which target and impair sensitive TCA cycle enzymes. Enzymes containing iron-sulfur clusters, such as aconitase, are particularly vulnerable; their impairment triggers a feedback loop of redox imbalances and increased oxidative stress (67). These disruptions significantly compromise cerebral function by reducing ATP synthesis, impeding synaptic maintenance, and facilitating the accumulation of neurotoxic metabolites.

### 4.4 Altered lipid metabolism and membrane remodeling

Beyond glucose impairment, a growing body of epidemiological and clinical evidence links disrupted lipid metabolism to the pathophysiology and progression of AD. Elevated concentrations of free fatty acids (FFAs) and their metabolic intermediates, such as acyl-carnitines and acyl-CoA, exert neurotoxic effects, leading to mitochondrial uncoupling and severe bioenergetic dysfunction (68). Analysis of cerebrospinal fluid (CSF) in AD patients reveals a significant increase in total FFA levels, although individual lipid species show distinct patterns of alteration (69).

Emerging research indicates that defects in lipid metabolism specifically affect the integrity of lipid rafts and the formation of lipid droplets. In the prodromal and early stages of Alzheimer's disease, researchers have documented significant changes in fatty acid composition within lipid rafts, along with a marked increase in cerebral lipid peroxidation (70). These structural changes likely contribute to the instability of neuronal membranes and signaling pathways.

A characteristic feature of the AD brain is the reduction of unsaturated fatty acids, particularly omega-3 polyunsaturated fatty acids (PUFAs) and monounsaturated fatty acids (MUFAs) such as oleic acid. Specifically, levels of docosahexaenoic acid (DHA)—the most abundant PUFA in the central nervous system—are significantly reduced in the hippocampus and cortex of AD patients (68, 71). This depletion leads to a lower overall unsaturation index, which strongly correlates with cognitive impairment. In contrast, even-chain saturated fatty acids are notably increased in the CSF, further reflecting the systemic metabolic shift associated with neurodegeneration (72).

### 4.5 Energetic deficits and synaptic failure

Synaptic disruption is a primary pathogenic hallmark of Alzheimer's disease, appearing long before widespread neurodegeneration. Epidemiological data show a strong correlation between synapse loss and cognitive decline, indicating that maintaining synaptic integrity is a central to disease progression (73). This dysfunction includes structural and functional changes in the presynaptic terminal, synaptic cleft, and postsynaptic dendrites. The failure of synaptic transmission is caused by multiple factors, including reduced mitochondrial energy supply at the nerve terminal and abnormal synaptic pruning mediated by microglial phagocytosis. Evidence from murine models shows that the accumulation of pathogenic Tau leads to significant synaptic loss and dysregulation even without extensive neuronal death (74).

Current research increasingly highlights that soluble Tau species, particularly oligomers, are more synaptotoxic than insoluble neurofibrillary tangles (NFTs) (75). This shift in understanding underscores the importance of targeting early-stage Tau aggregates rather than late-stage pathological inclusions to preserve cognitive function.

## 5 Molecular interactions between amyloid, tau and mitochondrial failure

### 5.1 Amyloid β-induced mitochondrial toxicity

**Figure 2:**
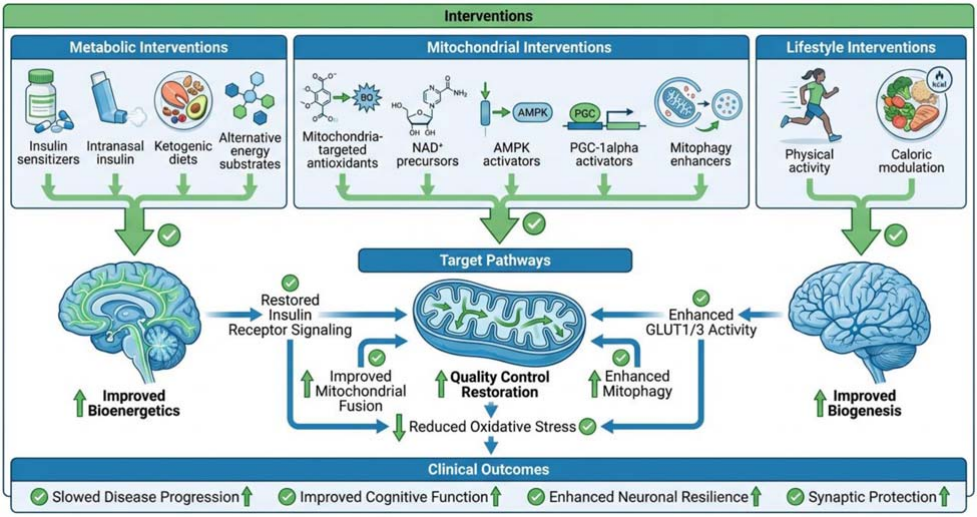
Therapeutic targeting of metabolic and mitochondrial dysfunction in Alzheimer's disease.

The accumulation of insoluble Aβ in Alzheimer's disease results from a fundamental imbalance between peptide synthesis and clearance. While familial AD, a less prevalent form, is driven by genetic mutations affecting $A\beta$ metabolism, most sporadic AD cases are attributed to inefficient Aβ clearance (76) (Figure 2).

Both insoluble fibrils and soluble Aβ oligomers exert potent neurotoxicity by forming plaques and triggering pathological cascades, including synaptic failure, excitotoxicity, changes in membrane permeability, and chronic inflammation (77, 78). The relationship between Aβ and mitochondria is bidirectional and self-reinforcing. Early Aβ accumulation directly impairs mitochondrial function, while mitochondrial dysfunction increases the production of mitochondria-derived reactive oxygen species (ROS). These ROS further stimulate Aβ production by activating β- and γ-secretases, creating a deleterious feed-forward loop that accelerates neuronal loss (79).

A hallmark of early AD is systemic impairment of cerebral energy metabolism (304). High ROS concentrations cause molecular damage both at the site of generation and in adjacent cellular structures (80). The activities of key metabolic enzymes—including cytochrome c oxidase (Complex IV), the pyruvate dehydrogenase complex, isocitrate dehydrogenase, α-ketoglutarate dehydrogenase, and ATP synthase—are significantly reduced in the AD brain. Notably, while these enzymes decline, the activities of succinate dehydrogenase (Complex II) and malate dehydrogenase often increase. This enzymatic imbalance compromises the inner mitochondrial membrane potential and severely reduces ATP production (80).

The accumulation of mitochondrial Aβ is not simply a passive byproduct of disease; it involves the targeting of specific proteins critical to organelle integrity. Key identified targets include Cyclophilin D (CypD), Aβ-binding alcohol dehydrogenase (ABAD), and human Presequence Protease (hPreP) (81). It has been shown that CypD can form complexes with Aβ within the mitochondria of cortical neurons in APP transgenic mice, increasing the translocation of CypD from the matrix to the inner membrane (82), an essential process in the opening of the mPTP (83). This causes dissipation of the internal membrane potential and generation of ROS, followed by rupture of the external membrane and non-specific release of intermembrane space proteins into the cytosol, which activate various signal transduction pathways such as apoptosis (84). The convergence of Aβ and ROS at the mPTP node serves as a critical checkpoint, shifting the cell from metabolic stress to programmed apoptosis.

Further exacerbating this dysfunction is the interaction between Aβ and ABAD, a mitochondrial matrix enzyme that uses NAD^+^ or NADH as a cofactor for the oxidation and reduction of alcohol groups (85). Aβ-ABAD complexes have been detected in both transgenic mouse models and human AD brains. This pathological interaction prevents NAD^+^ binding, thereby impairing mitochondrial enzymatic activity and directly contributing to the cognitive deficits observed during AD progression (83).

### 5.2 Tau mediated mitochondrial transport disruption

While the exact mechanisms by which aberrant Tau triggers mitochondrial dysfunction are still being elucidated, its impact on synaptic integrity is well documented. Synaptic mitochondria are essential for meeting the high bioenergetic demands of the nerve terminal and maintaining calcium homeostasis. Consequently, disruption of mitochondrial transport to the synapse is a primary driver of synaptic degeneration and eventual neuronal death (67).

Evidence from cellular and murine models indicates that Tau overexpression and hyperphosphorylation severely disrupt the localization and distribution of mitochondria (86). This displacement causes profound axonal dysfunction and synaptic loss (87). Recent studies using induced pluripotent stem cells (iPSCs) from patients with frontotemporal dementia (FTD) carrying the R406W Tau mutation have confirmed these trafficking impairments. In cerebral organoids derived from these iPSCs, axonal mitochondria showed decreased stability and a shift toward retrograde orientation, resulting in a net reduction of mitochondrial density within the axon (88).

Similarly, iPSC-derived neurons with the N279K and P301L Tau mutations show a significant reduction in anterograde axonal transport compared to healthy controls (89). This failure in the forward delivery of mitochondria—caused by Tau interfering with motor proteins such as kinesin—deprives the distal synapse of essential ATP, making it vulnerable to metabolic collapse.

### 5.3 ROS-driven amplification loops

Mitochondrial dysfunction is an early-stage hallmark of AD, characterized by diminished ATP synthesis and a reciprocal surge in reactive oxygen species (ROS) (89). Beyond bioenergetic failure, Aβ peptides directly compromise mitochondrial integrity by destabilizing mitochondrial membranes and inhibiting the respiratory chain, further exacerbating oxidative stress (90).

The impairment of mitochondrial function leads to the initiation of a subsequent inflammatory reaction. Molecules released from dysfunctional mitochondria, including mitochondrial DNA (mtDNA) and cytochrome c, serve as damage-associated molecular patterns (DAMPs). These DAMPs stimulate Pattern Recognition Receptors (PRRs) and the NLRP3 inflammasome, promoting persistent neuroinflammation (91).

Crucially, neuronal homeostasis relies on the structural and functional tethering of the endoplasmic reticulum (ER) and mitochondria via Mitochondria-Associated Membranes (MAMs). These contact sites regulate calcium signaling, lipid synthesis, and protein folding. In Alzheimer's disease (AD), the disruption of MAMs enhances inflammatory signaling and impairs the cellular response to ER stress (92). These structural alterations ultimately dictate cell fate by creating a feedback loop of elevated reactive oxygen species (ROS) production and a diminished antioxidant capacity to neutralize neurotoxic metabolites (93).

### 5.4 Mitochondrial dysfunction as an upstream driver of amyloid and tau pathology

A significant shift in Alzheimer's disease (AD) research is represented by the Mitochondrial Cascade Hypothesis, which posits that mitochondrial dysfunction is the primary driver of AD pathophysiology, rather than a secondary consequence of Tau or Aβ accumulation (94). This theory suggests that age-related mitochondrial decline is the fundamental catalyst for the disease. According to this model, an individual's baseline mitochondrial function is determined by genetic inheritance, while the rate of age-associated mitochondrial decline is influenced by environmental factors. The interaction of these two variables determines the age of onset and the rate of disease progression.

Importantly, this theory posits that the production of Aβ is influenced by mitochondrial dysfunction triggered by toxins. Consequently, the buildup of Aβ, which initiates well in advance of the manifestation of symptoms, is viewed as an indicator of aging and metabolic challenges in the brain rather than the root cause (94).

### 5.5 The bidirectional cascade: crosstalk model

There has been much discussion and modification of a model that describes how AD biomarkers grow in connection to one another as well as to the beginning and progression of clinical symptoms (95). It is important to acknowledge that tau and Aβ pathology may start separately in LOAD. The dual-pathway concept posits that Aβ elevation and tau hyperphosphorylation may be independent pathophysiological processes with harmful synergy. According to the Aβ-tau interaction hypothesis, Aβ and tau pathology, which results in synaptic dysfunction, neuronal death, and cognitive impairment (96), is determined by the intrinsic interaction with Aβ and tau (Aβ42 enhances tau phosphorylation, truncation, and aggregation, while tau can increase Aβ synthesis). The impacts of several risk factors and biomarkers, including as tau hyperphosphorylation and Aβ elevation, may be caused by impaired neuroplasticity and mitochondrial dysfunction (97), which may be a common upstream etiology of AD.

## 6 Neuroinflammation and oxidative stress in bioenergetics failure

### 6.1 Microglial activation and metabolic rewiring

**Figure 3:**
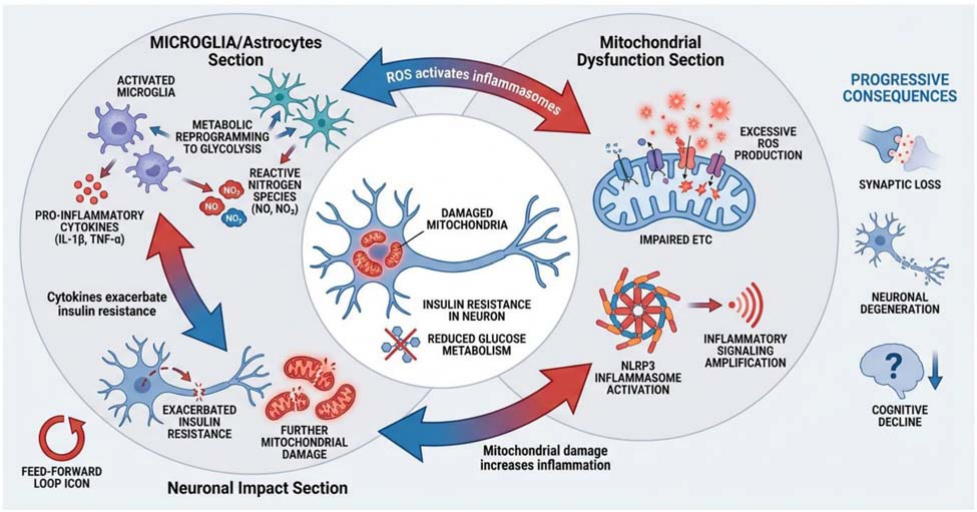
Neuroinflammation, metabolic reprogramming, and mitochondrial crosstalk.

Microglia, the indigenous immune cells of the central nervous system, are part of the myeloid lineage and play a crucial role in protecting the brain. They detect harmful stimuli through Pattern Recognition Receptors (PRRs) by recognizing pathogen-associated molecular patterns (PAMPs) and damage-associated molecular patterns (DAMPs) (98) (Figure 3). Initially, the activation of microglia is beneficial for protecting the brain; Damage-Associated Molecular Patterns (DAMPs) such as Aβ species are cleared by microglia to prevent the formation of plaques (99). However, prolonged activation can lead to chronic inflammation and a harmful change in function. In the presence of ongoing accumulation of Aβ, a subset of microglia becomes abnormally activated, causing a neurotoxic effect that accelerates the progression of neurodegenerative diseases (100, 101).

Recent research suggests that this change in function is driven by changes in metabolism. Studies conducted in laboratory settings show that exposure to Aβ leads to a shift from using oxidative phosphorylation to aerobic glycolysis, similar to the Warburg effect. As the disease progresses, the development of immune tolerance in microglia toward Aβ plaques becomes a key feature (102). Despite the initial shift toward glycolysis, microglia in advanced stages of Alzheimer's disease experience a decline in both glycolytic and oxidative phosphorylation pathways, resulting in a state of “energetic exhaustion” that impairs their ability to clear debris and protect neurons (103).

### 6.2 Astrocytic dysfunction and lactate transport failure

Neurons primarily rely on mitochondrial oxidative phosphorylation for energy production, while astrocytes have a unique metabolic profile with high glycolytic activity. Astrocytes express high levels of glycogen and key enzymes involved in glycolysis, making them particularly responsive to the changing energy needs of the brain (21).

A crucial aspect of this metabolic collaboration is the Astrocyte-Neuron Lactate Shuttle (ANLS). When astrocytes undergo glycolysis, glucose is quickly converted into L-lactate, which is then transported out of the cells through monocarboxylate transporters (MCTs)—specifically MCT1 and MCT4 on astrocytes and MCT2 on neurons. Once taken up by neurons, L-lactate is converted back to pyruvate to support the Tricarboxylic Acid (TCA) cycle (20). The shuttle system is not just an additional pathway; it is crucial for complex neuronal functions like long-term potentiation (LTP) and memory consolidation. Any interruption in astrocytic glycolysis or the efficiency of MCT transport directly impacts neuronal survival. In Alzheimer's disease, dysfunction in the ANLS is a key metabolic obstacle that plays a significant role in early cognitive decline (20).

### 6.3 NADPH oxidase activation and oxidative burst

Memory loss is a key characteristic of Alzheimer's disease (AD), a neurodegenerative condition caused by a complex pathological process that involves the overproduction of reactive oxygen species (ROS) (104). This oxidative imbalance in the brain is due to a dual mechanism: an increase in pro-oxidant enzymes and a decrease in antioxidant defenses like catalase and superoxide dismutase (SOD) (Table 2).

**Table 2:** Key metabolic alterations observed in Alzheimer's disease pathophysiology.

**Metabolic alteration**	**Cellular level affected**	**Mechanistic consequence**	**Impact on disease progression**	**References**
Reduced cerebral glucose uptake	Neurons	Energy deficit	Early cognitive impairment	(286)
Insulin resistance	Neurons, astrocytes	Impaired signaling	Synaptic dysfunction	(287)
GLUT1 downregulation	Blood–brain barrier	Limited glucose transport	Regional hypometabolism	(288)
GLUT3 reduction	Neurons	Reduced neuronal glucose use	Memory decline	(289)
Impaired glycolysis	Cytosol	Reduced pyruvate supply	Mitochondrial failure	(290)
Altered lactate shuttling	Astrocyte–neuron axis	Energy uncoupling	Synaptic vulnerability	(291)
Decreased TCA cycle flux	Mitochondria	Reduced ATP	Neuronal degeneration	(292)
NAD^+^ depletion	Mitochondria	Impaired redox balance	Accelerated aging	(293)
Increased lipid peroxidation	Cell membranes	Oxidative damage	Neuronal loss	(294)
Ketone utilization deficit	Neurons	Limited alternative fuel	Advanced AD	(295)
Mitochondrial calcium overload	Mitochondria	Apoptosis activation	Cell death	(296)
Reduced ATP synthesis	Mitochondria	Energy failure	Cognitive decline	(297)
Impaired insulin receptor signaling	Synapses	Plasticity loss	Learning deficits	(298)
Increased ROS generation	Mitochondria	Oxidative stress	DNA damage	(305)
Altered amino acid metabolism	Neurons	Neurotransmitter imbalance	Behavioral changes	(299)
Disrupted cholesterol metabolism	Neurons	Membrane instability	Amyloid aggregation	(296)
Metabolic inflexibility	Brain networks	Poor stress adaptation	Disease progression	(293)

One of the main enzymes responsible for producing superoxide during long-term neuroinflammation is Nicotinamide adenine dinucleotide phosphate oxidase (NADPH oxidase). This complex, which is located on cell membranes and consists of multiple subunits, plays a significant role in the development of Alzheimer's disease (AD) and Parkinson's disease (PD). In these conditions, the persistent activation of NADPH oxidase in microglia leads to continuous oxidative stress and damage (105).

The activation of phagocytic NADPH oxidase is a tightly controlled process that occurs in specific locations within the cell. When stimulated, the cytosolic components of the enzyme, such as p47phox, p67phox, and p40phox, move to the cell membrane to interact with the membrane-bound subunits, gp91phox and p22phox. The key component of this complex is the NOX2 subunit, which facilitates the transfer of an electron from NADPH to molecular oxygen (O2), resulting in the production of the superoxide radical anion (O2–). In the context of Alzheimer's disease, the excessive and uncontrolled generation of superoxide by microglial NADPH oxidase contributes to significant oxidative damage, directly contributing to neurodegeneration and cognitive impairment (106).

### 6.4 Inflammatory cytokines and mitochondrial damage

Dysregulated microglial function plays a key role in various neurodegenerative, autoimmune, and viral diseases, such as Alzheimer's and Parkinson's disease. When amyloid beta interacts with microglial receptors, it triggers an inflammatory response characterized by the release of pro-inflammatory cytokines like TNF-alpha, IL-1beta, and IL-6 (107). This inflammatory activation is driven by increased levels of inducible nitric oxide synthase (iNOS) and NADPH oxidase (NOX), leading to the production of reactive oxygen species (ROS). These factors create a persistent inflammatory environment that contributes to neuronal damage and loss of synapses over time (108).

The metabolic and inflammatory status of microglia is closely tied to their mitochondrial health. In the context of Alzheimer's disease (AD), both microglia and neighboring neural cells display a range of mitochondrial abnormalities, including:

1. Genetic instability: The accumulation of mitochondrial DNA (mtDNA) mutations with age, which can trigger inflammation by acting as damage-associated molecular patterns (DAMPs).
2. Bioenergetic dysfunction: Changes in membrane potential, reduced ATP production, and disruptions in the Electron Transport Chain (ETC).
3. Structural irregularities: Increased mitochondrial fragmentation and impaired mitophagy, the process responsible for removing damaged organelles (109, 305).

## 7 Systemic metabolic disorders that contribute to Alzheimer's disease

### 7.1 Diabetes, insulin resistance and metabolic syndrome

The complex interplay of diabetes, dyslipidemia, obesity, and hypertension in clinical settings makes it challenging to pinpoint their individual roles in pathology. Research often concentrates on one factor, neglecting the combined impact of these risk factors on Alzheimer's disease (AD) risk. Therefore, a more inclusive strategy involves examining metabolic syndrome, a collection of conditions such as central obesity, hypertension, hyperlipidemia, and diabetes, as a comprehensive risk profile (110).

Numerous epidemiological studies have investigated the association between diabetes mellitus (DM) and Alzheimer's disease (AD), with a significant body of evidence indicating a direct connection between Type 2 Diabetes (T2D) and an elevated risk of developing AD. Various mechanisms have been proposed to elucidate this relationship, including insulin deficiency, central insulin resistance, impaired insulin receptors, and neurotoxicity induced by high blood sugar levels. Additionally, the harmful effects of advanced glycation end products (AGEs), chronic neuroinflammation, and damage to the brain's blood vessels contribute to the progression of the disease (111).

The rising occurrence of Alzheimer's disease (AD) in individuals with type 2 diabetes (T2D) and obesity suggests common underlying molecular pathways (112). Specifically, impaired insulin signaling plays a crucial role in accelerating AD pathology (113). The insulin-degrading enzyme (IDE) is responsible for clearing both insulin and Aβ, a key protein in AD. However, in cases of hyperinsulinemia due to insulin resistance, IDE can become overwhelmed, prioritizing insulin degradation over Aβ breakdown. This leads to reduced clearance of Aβ and its accumulation in the brain. Additionally, IDE activity, a significant risk factor for sporadic AD, naturally decreases with age, further compromising the brain's ability to break down proteins (114).

### 7.2 Obesity, dyslipidemia, and chronic low grade inflammation

The connection between excess body fat and cognitive decline, previously overlooked, is now supported by a strong body of epidemiological evidence. Meta-analytical studies show that individuals with obesity or related metabolic conditions are almost twice as likely to develop Alzheimer's disease (AD) (115). Elevated levels of systemic cholesterol, a common feature of obesity, have a significant impact on the production of Aβ proteins. Although the brain primarily produces its own cholesterol and isolates it from the rest of the body, research suggests that prolonged high cholesterol levels in the body may promote the transfer of cholesterol across the blood-brain barrier (BBB), contributing to the development of amyloid plaques (116).

A key process that connects metabolic issues in the body to brain-related problems is the movement of Aβ from the bloodstream into the central nervous system (CNS). This transfer is mainly facilitated by the receptor for advanced glycation end products (RAGE). When levels of Aβ in the blood remain high for an extended period, it enhances the transport of Aβ into the brain through RAGE. Once in the brain, Aβ interacts with neurons, leading to a series of cellular stress responses and neurotoxic effects (117).

The timing of obesity plays a crucial role in determining the risk of developing dementia. Studies based on population cohorts indicate that reducing midlife obesity by 20% could potentially lower the prevalence of dementia in older individuals by 10% (118). Interestingly, there is a phenomenon known as the “obesity paradox” where having a higher Body Mass Index (BMI) in late life is associated with a lower risk of dementia, despite midlife obesity being a significant risk factor. This paradox may be attributed to the unintended weight loss that occurs during the early stages of dementia (119).

Obesity is essentially a condition characterized by chronic, low-grade inflammation due to the enlargement and increased number of fat cells (120). The excess fat tissue releases inflammatory substances such as TNF-alpha, IL-6, MCP-1, and various adipokines, which attract more immune cells and sustain inflammation. Recent studies suggest that this inflammation in fat tissue is the main cause of cognitive decline in obese individuals (121). This inflammatory environment triggers the formation of inflammasome complexes, which intensify the immune response locally and throughout the body, ultimately affecting the brain's neuro-immune system (122).

### 7.3 Cardiovascular and cerebrovascular comorbidities

The heart-brain axis is a complex system of pathological mechanisms that connect the circulatory and cerebrovascular systems. Cardiovascular disease (CVD) and Alzheimer's disease (AD) are common chronic conditions associated with aging, and their increasing prevalence is thought to be linked to abnormalities within this axis. Although the specific molecular regulators are still being studied, it is now understood that established risk factors for CVD play a significant role in the development of AD-related dementia (123, 124).

Both experimental models and long-term observational cohorts have confirmed the link between Alzheimer's disease (AD) and cardiovascular health. Specifically, levels of amyloid-beta (Aβ 1–40) in the bloodstream are linked to the presence of coronary artery disease, irrespective of cognitive function in individuals (125). This indicates that the dynamics of amyloid in the body may be a shared factor in both heart and brain dysfunction.

The connection between Alzheimer's disease (AD) and atherosclerosis has been a topic of discussion for many years. Recent research indicates that individuals with AD have a notably higher occurrence of cerebrovascular atherosclerosis in comparison to individuals of the same age who do not have AD (126, 127). The strong association between Alzheimer's disease (AD) and atherosclerosis in the Circle of Willis, the main arterial network that supplies the brain, is of great importance. Research based on population studies has shown that structural abnormalities in this crucial vascular junction can lead to reduced cerebral blood flow and damage to the blood-brain barrier. This, in turn, accelerates the buildup of Aβ protein and the advancement of neurodegenerative processes (128).

### 7.4 Gut microbiota dysbiosis and metabolic-endocrine crosstalk

The gradual buildup of abnormal protein aggregates in the central nervous system (CNS) is a key characteristic of neurodegenerative diseases such as Alzheimer's disease (AD), Parkinson's disease (PD), and amyotrophic lateral sclerosis (ALS) (129). It is noteworthy that the age-old Hippocratic belief that “all disease originates in the gut” remains relevant in modern neurobiology, with growing evidence supporting the importance of the gut-brain axis in maintaining neural well-being (130).

One key characteristic of gut dysbiosis in Alzheimer's disease is an increased ratio of Firmicutes to Bacteroidetes (F/B ratio). This imbalance in gut bacteria is connected to the buildup of amyloid precursor protein (APP) in the intestines at the onset of the disease (131). Studies using APP/PS1 mouse models have shown that changes in gut microbiota composition can lead to higher levels of amyloid beta (Aβ) in the central nervous system, as well as notable declines in memory and spatial learning abilities (132).

The release of bacterial metabolites provides a possible link between the gut and the brain. Short-chain fatty acids (SCFAs) have been found to disrupt the interactions between proteins that are crucial for the assembly of Aβ, potentially offering a protective effect on the brain (133). On the other hand, the microbial metabolite trimethylamine N-oxide (TMAO) has been associated with the pathophysiology of Alzheimer's disease (134). TMAO worsens Aβ pathology by increasing the activity of β-secretase, leading to faster amyloid formation and cognitive decline. Additionally, TMAO affects the release of calcium from cellular stores in response to stimuli, promoting excessive platelet reactivity. This mechanism facilitates the spread of Aβ from platelets into the bloodstream and eventually into the brain tissue, adding to the overall amyloid accumulation (131, 135).

## 8 Clinical biomarkers of mitochondrial and metabolic dysfunction

### 8.1 Imaging biomarkers

In the clinical evaluation of neurodegenerative disorders, particularly Alzheimer's disease (AD), the use of 18 F-fluorodeoxyglucose positron emission tomography (FDG-PET) plays a crucial role in aiding diagnosis. Traditionally, FDG-PET has been utilized to visualize synaptic loss, neuropil atrophy, and neuronal functional decline. Within the established research paradigm, decreased FDG uptake has been classified as an “N” (neurodegeneration) biomarker, indicating reduced metabolism. However, according to recent research, FDG-PET signals may not only reflect neuronal activity but also astrocytic glucose utilization through glutamate transport (GLT-1). This leads to a reevaluation of metabolic imaging in AD; in particular, by preserving glucose absorption levels, reactive astrogliosis may paradoxically “mask” underlying neuronal loss. Therefore, rather than being a stand-alone indicator of neuronal health, FDG-PET should be viewed as a measurement of the larger neurovascular unit (136).

Recent research suggests that decreased FDG uptake could be indicative of vascular abnormalities, particularly those related to dysfunction in the blood-brain barrier (BBB). Studies conducted within the Alzheimer's Disease Neuroimaging Initiative (ADNI) cohort have examined the need to redefine FDG-PET as a distinct biomarker for metabolic dysfunction, denoted as “F” (representing FDG hypometabolism). This reclassification would separate it from the general “N” category in individuals who are amyloid-positive and tau-positive, allowing for a more detailed understanding of metabolic impairment that is independent of structural atrophy (126).

Magnetic Resonance Spectroscopy (MRS) offers a direct and non-invasive method to examine the bioenergetic and metabolic abnormalities linked to neurodegenerative conditions. *In vivo* 1H-MRS enables the measurement of various important metabolites, including:

- N-acetylaspartate (NAA): a marker of neuronal health.
- Myo-inositol (mI): an indicator of astrogliosis and neuroinflammation.
- Choline-containing compounds (Cho): reflecting membrane turnover.
- Creatine (Cr) and Phosphocreatine (PCr): representing the brain's energy reserves.

While these metabolites exhibit distinct peaks in the spectrum, others like Glutathione (GSH), GABA, glucose, and lactate display more subtle and overlapping signals, leading to increased measurement variability (137).

Meta-analyses have consistently found a metabolic profile characterized by decreased N-acetylaspartate (NAA) and increased myo-inositol (mI) levels in both Mild Cognitive Impairment (MCI) and Alzheimer's Disease (AD) (138, 139). However, many studies have focused exclusively on proton magnetic resonance spectroscopy (1H-MRS) and have not incorporated markers of oxidative stress (OS) and neuroinflammation. As a result, there is still a gap in diagnostic knowledge, as it remains uncertain whether specific longitudinal metabolic changes can accurately predict the progression from MCI to AD or provide insights into the underlying molecular mechanisms.

### 8.2 Blood and CSF biomarkers

Once considered a mere waste product of metabolism, L-lactate is now acknowledged for its various physiological functions in the central nervous system, such as modulating neuronal activity and ensuring energy balance (20). Changes in cerebrospinal fluid (CSF) lactate levels are important clinical markers of metabolic dysfunction; for example, increased CSF lactate is a key feature of energy metabolism impairment in mitochondrial disorders and hepatic encephalopathy (140, 141).

Alzheimer's disease is primarily characterized by impaired glycolytic pathways and mitochondrial dysfunction. Extensive clinical studies have shown that individuals with Alzheimer's disease have notably elevated levels of cerebrospinal fluid lactate (142). Interestingly, higher lactate levels are frequently observed in patients with milder cognitive impairment, indicating a potential compensatory metabolic change or a shift resembling the Warburg effect in the early stages of neurodegeneration. Additionally, there is a negative association between cerebrospinal fluid lactate levels and Tau protein levels, suggesting a possible connection between anaerobic metabolic shifts and cytoskeletal abnormalities.

Mitochondrial dysfunction directly affects the brain's main process for metabolizing glucose, known as the Tricarboxylic Acid (TCA) cycle. Abnormalities in the TCA cycle, along with reduced overall metabolism, result in a failure to produce ATP effectively (143, 144). While the exact sequence of events is still debated, evidence suggests that the buildup of Aβ disrupts mitochondrial function, leading to a breakdown in energy production that hinders synaptic plasticity and cognitive abilities (145).

**Figure 4:**
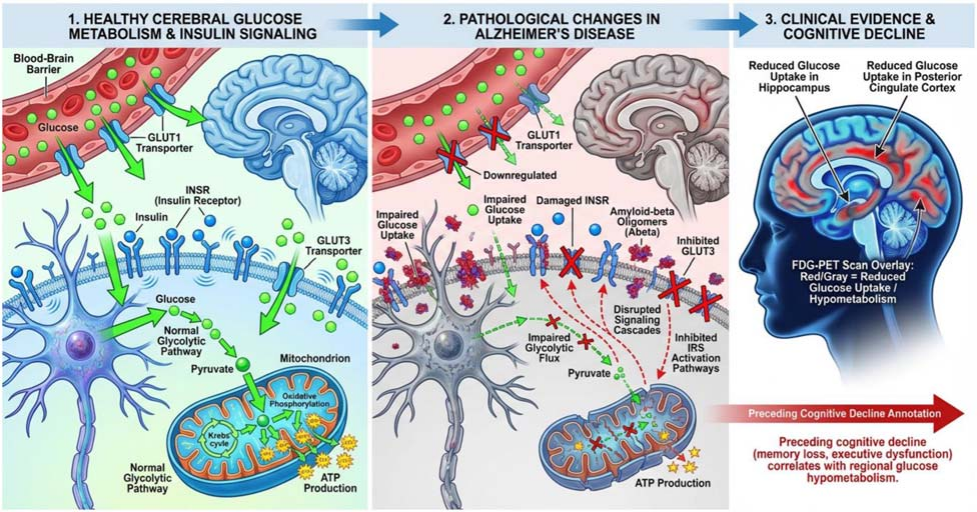
Cerebral glucose hypometabolism and insulin resistance in Alzheimer's disease.

Mitochondrial DNA copy number (mtDNA-CN) is a valuable indicator of mitochondrial functional capacity and genome stability. Abnormal mtDNA-CN levels can indicate issues with the transcription machinery and replication of mitochondrial DNA (146). In individuals with Alzheimer's disease (AD), pyramidal neurons show a significant decrease in mitochondrial DNA copy number (mtDNA-CN) compared to those without cognitive impairment (147). This reduction is also evident in peripheral blood mononuclear cells of both AD and Mild Cognitive Impairment (MCI) patients (148). These findings suggest that mtDNA-CN could be a valuable preclinical biomarker for mitochondrial dysfunction, potentially offering a target for therapeutic interventions to restore genomic stability in the mitochondria (149) (Figure 4).

Table 3 summarizes the relationship between particular metabolic dysfunctions, their clinical readouts, and the accompanying therapeutic implications in order to give a coherent overview of how these varied markers align with the metabolic-pathophysiological framework of AD.

**Table 3:** Integrative framework of pathology, biomarkers and the therapeutic interventions.

**Pathology**	**Biomarkers**	**References**	**Therapeutic intervention**
Hypometabolism in posterior cingulate and parietotemporal cortices.	18 F-fluorodeoxyglucose positron emission tomography (FDG-PET)	(300)	Neuroprotective effects of GLP 1 RAs and spermidine is the stimulation of autophagy and mitophagy.
High lactate levels in MCI, Warburg like effects due to mitochondrial decay and negative coorelation with Tau protein.	CSF-L Lactate	(142)	Mitophagy enhancers (Spermidine) to rescue mitochondria.
Genomic Instability: significant decrease in pyramidal neurons and peripheral blood mononuclear cells.	Mitochondrial DNA copy number (mtDNA-CN)	(147) (148)	Mitochondrial biogenesis stimulators: (e.g., PGC-1alpha activators, NAD+ precursors, or Spermidine) to restore mitochondrial density and ATP production.
Loss of Neuronal Integrity: Decreased N-acetylaspartate (NAA) and increased myo-inositol (mI) levels in both Mild Cognitive Impairment (MCI) and Alzheimer's Disease.	Magnetic Resonance Spectroscopy 1H-MRS	(139)	Mitochondrial targeted antixoidants such as MitoQ, SkQ1 for neuronal viability.

### 8.3 Metabolomics and lipidomics signatures

Metabolomics plays a crucial role in identifying biomarkers for neurodegenerative disorders by allowing for the simultaneous quantification of thousands of metabolites in different organ systems. Recent studies have highlighted specific profiles of bile acids (BA), amino acids (AA), and fatty acids (FA) as potential indicators for Alzheimer's disease (AD) and Mild Cognitive Impairment (MCI) (150, 151). While previous research has focused on low-molecular-weight metabolites, lipid metabolites, which have significant biological activity in the central nervous system, are now gaining recognition in these investigations.

Lipidomics, a specialized field within metabolomics, is dedicated to the thorough identification and characterization of various lipid classes, subclasses, and molecular species. By combining metabolomics with lipidomics, scientists can create a comprehensive overview of the metabolic landscape, offering a valuable tool to investigate the underlying mechanisms of Traditional Chinese Medicine (TCM) and other therapeutic approaches. As the brain is the organ with the highest concentration of lipids in the body, lipids play a crucial role in maintaining the structural integrity of cell membranes and regulating important signaling pathways. Changes in lipid metabolism are closely associated with disruptions in homeostasis and the development of neurodegenerative diseases.

A significant study in lipidomics identified a set of 26 sphingolipids and glycerophospholipids that can effectively differentiate between patients with Alzheimer's disease (AD) and cognitively normal individuals (152). Specifically, elevated concentrations of certain sphingomyelins (SMs) such as SM C16:0, SM C18:1, SM C16:1, and SM (OH) C14:1 were observed in AD patients. These lipid profiles are closely associated with the severity of the disease, indicating their potential utility as precise molecular markers for diagnostic and therapeutic purposes.

### 8.4 Genetic biomarkers

While around 75 genetic loci have been associated with an elevated risk of Alzheimer's disease (AD), most cases are sporadic or late-onset variants. Among these, the Apolipoprotein E (APOE) gene, particularly the ε4 allele (APOE4), stands out as the most significant and consistent genetic risk factor. In the last 30 years, close to 10,000 studies have explored the complex connection between APOE and AD pathology (153).

The APOE genotype plays a crucial role in clinical research and trials, impacting prognosis, risk evaluation, and treatment planning. Particularly, the APOE genotype has a notable effect on how individuals respond to anti-amyloid monoclonal antibodies like lecanemab, donanemab, and aducanumab. This genetic interaction has led to the creation of therapies that target the specific disease pathways influenced by the APOE4 protein (154, 155).

Data from long-term studies, like the Alzheimer's Disease Neuroimaging Initiative (ADNI), show that the APOE4 gene variant is a strong indicator of both amyloid buildup and shrinkage of the hippocampus, even in the early stages of Alzheimer's disease. Including APOE status in longitudinal analysis enables more accurate identification of when symptoms may appear and helps pinpoint individuals who are at higher risk before they show any symptoms (156).

In addition to nuclear genetics, variations in mitochondrial DNA (mtDNA) - categorized into established haplogroups - are known to play a significant role in the susceptibility to Alzheimer's disease (AD) (157). A human mitochondrial haplogroup is characterized by a specific set of mutations that have accumulated along a maternal lineage (158). These haplogroups are identified by letters (e.g., H, V, L) and further refined by alphanumeric suffixes (e.g., H51A1). Research indicates that certain haplogroups, such as U, H, and J, may act as significant risk factors for AD, potentially affecting the efficiency of oxidative phosphorylation and production of reactive oxygen species (ROS) (157, 159).

## 9 Emerging therapeutic interventions targeting metabolism and mitochondria

### 9.1 Metabolic modulators

#### 9.1.1 Intranasal insulin

Central nervous system (CNS) insulin deficiency may result from impaired insulin transport across the blood-brain barrier (BBB). Accordingly, increasing cerebral insulin levels has been proposed as a strategy to halt the neurodegenerative processes associated with Alzheimer's disease (AD) (160). This therapeutic rationale has driven investigations into various pharmacological agents and their delivery methods. Insulin receptors are most densely concentrated in the olfactory bulb, cerebral cortex, hippocampus, hypothalamus, cerebellum, and choroid plexus (161). Leveraging this distribution, insulin can be administered to the brain via the olfactory and trigeminal nerve pathways. This intranasal delivery route enables insulin to bypass the blood-brain barrier by crossing the nasal epithelium and directly accessing the central nervous system (162). At the molecular level, insulin binding induces autophosphorylation of the insulin receptor, followed by activation of the insulin receptor substrate (IRS). Subsequent phosphorylation of IRS triggers the AKT signaling pathway, a critical downstream event linked to enhanced neuroprotection, improved synaptic plasticity, and better cognitive function in Alzheimer's disease patients (163).

Furthermore, the cognitive response to insulin appears to be influenced by sex-specific physiologic variables, albeit the evidence is mixed.In later tests, IN insulin injection before nocturnal sleep was found to promote word-pair acquisition in women while having the opposite effect in males (164). In an acute investigation that only included healthy male participants (165), IN insulin compared to placebo (diluent) boosted the odor-cued recall of spatial memory, while an inhibiting impact of IN insulin (vs. diluent) on olfactory sensitivity was detected in young healthy women but not males (166). These disparate findings highlight the importance of sex-stratified analysis in metabolic AD trials.

#### 9.1.2 GLP-1 receptor agonists

Glucagon-like peptide-1 (GLP-1) is an incretin hormone predominantly secreted by intestinal L-cells and is endogenously produced by neurons within the central nervous system (CNS) (167). The GLP-1 receptor (GLP-1R) is extensively expressed in multiple brain regions, including the hippocampus, neocortex, hypothalamus, and cerebellum (168). Although GLP-1R is constitutively expressed by neurons, its expression in glial cells is generally induced only during active inflammatory responses (169). Recent animal model studies have demonstrated that GLP-1 and its agonists exert notable neuroregulatory and neuroprotective effects (170).

The neurotrophic and neuroprotective effects of GLP-1 in the central nervous system are mediated by its interaction with GLP-1R (167, 171). Within the nucleus tractus solitarius, activation of GLP-1R is believed to be regulated through the PKA pathway, which decreases AMPK phosphorylation. This signaling cascade contributes to appetite suppression, delayed gastric emptying, and weight reduction (172). Moreover, genetic modulation of this receptor highlights its critical role in neural function: deletion of GLP-1R markedly impairs cognitive performance, while its overexpression enhances cognitive abilities in murine models (173).

Although preclinical evidence suggests that GLP-1RAs can cross the blood-brain barrier (BBB), the efficacy of CNS delivery and the precise therapeutic dosage necessary to achieve neuroprotective concentrations in people are unclear. Furthermore, the heterogeneity in responses found across different transgenic AD mouse models—some of which exhibit no discernable effect on Aβ plaque burden (174), highlights the underlying complexity of the illness and implies a need for more personalized, precision-based therapy methods (175). Recent systematic reviews and meta-analyses of randomized controlled trials (RCTs) emphasize the translational problem. These investigations indicated that established GLP-1RAs, such as liraglutide and exenatide, did not have a substantial impact on core AD biomarkers (Aβ and tau) or generate noticeable cognitive benefits in the short term. These data imply that, while metabolic regulation remains a promising target, it may not be sufficient as a standalone treatment after advanced neurodegeneration has occurred (176).

#### 9.1.3 Metformin and AMPK activation

Metformin is widely recognized as the first-line therapy for Type 2 diabetes, endorsed by clinical guidelines for its excellent safety profile, high bioavailability, and weight-neutral properties (177). Its primary mode of action involves inhibiting hepatic gluconeogenesis and enhancing peripheral insulin sensitivity, leading to reduced blood glucose levels (178). In addition to its systemic metabolic advantages, metformin can penetrate the blood-brain barrier (BBB) and has shown promise in improving cognitive function. Recent research suggests that metformin may also impact the gut microbiota composition, potentially influencing the gut-brain axis in the context of Alzheimer's disease (AD) (179).

Experimental studies have shown that metformin treatment can improve cognitive performance in female mice with AβPP transgenic models (180). Interestingly, research using different genetic models of AMPK activation suggests that hepatic AMPK signaling could potentially alleviate cognitive impairments associated with Alzheimer's disease, although the specific mechanisms involved are still being investigated (181). Importantly, comparisons between male and female mice indicate that metformin may have a more significant positive impact on cognitive function in females, highlighting the importance of sex as a key factor in determining the drug's therapeutic benefits (181).

Metformin treatment has been found to have a positive impact on hippocampal health by supporting the generation of new neurons and maintaining cognitive abilities. This neuroprotective effect is believed to be achieved through the inhibition of the amyloidogenic pathway and reduction of neuroinflammation. The underlying mechanism involves the regulation of the AMPK/mTOR/S6K/BACE1 signaling pathway, which controls the production of Aβ and the inflammatory response in brain cells (182).

Long-term metformin medication has been linked to the development of Alzheimer's disease (183). A prospective research of 732 Korean T2DM patients followed for more than 9 years found that long-term metformin medication was associated with cognitive decline and an elevated risk of AD (184). A systematic review and meta-analysis revealed that metformin medication is associated with the development of Alzheimer's disease (AD) (185). The underlying mechanism for the development of AD after long-term metformin usage is connected to vitamin B12 insufficiency (186). A cohort study of T2DM patients (*n* = 126) and non-T2DM (*n* = 1,228) found that cognitive dysfunction in T2DM patients on metformin therapy is associated with low B12 serum levels (186). As a result, B12 supplements may help to prevent or reduce metformin-induced B12 deficiency and the development of Alzheimer's disease. Vitamin B12 deficiency is detected in 4.3% of patients using metformin (187).

### 9.2 Mitochondrial targeted antioxidants

#### 9.2.1 MitoQ

MitoQ is a specialized antioxidant designed to specifically target the mitochondria in the context of Alzheimer's disease (AD). This compound is created by combining ubiquinone, an essential component of the mitochondrial electron transport chain, with a lipophilic triphenylphosphonium (TPP^+^) cation through a ten-carbon aliphatic chain. The TPP^+^ component helps MitoQ to accumulate preferentially in the mitochondrial matrix, driven by the mitochondrial membrane potential. Once inside the mitochondria, Complex II converts ubiquinone into its active antioxidant form, ubiquinol. In this state, MitoQ effectively combats oxidative stress by inhibiting lipid peroxidation and neutralizing reactive oxygen species (ROS).

Evaluation of MitoQ in various Alzheimer's disease (AD) experimental models has confirmed its strong reactive oxygen species (ROS)-scavenging abilities. In transgenic AD mouse models, early administration of MitoQ has been shown to significantly reduce amyloid beta (Aβ) peptide levels, decrease synaptic loss, and alleviate astrogliosis. These cellular enhancements are associated with the preservation and improvement of cognitive function, indicating that early intervention targeting mitochondria could be a promising therapeutic approach (188).

Preclinical safety tests suggest that MitoQ has a somewhat restricted treatment window. Preliminary studies in mouse models indicated a feasible dosage of 20 mg/kg, with considerable toxicity found at 27 mg/kg. Long-term, low-dose administration did not show such negative consequences, indicating that the observed toxicity is likely due to excessive TPP^+^ buildup. At large concentrations, the lipophilic TPP^+^ cation can accumulate in the mitochondrial matrix, disrupting the membrane potential and impairing organelle function. These findings emphasize the importance of accurate pharmacological titrations for transferring mitochondrial antioxidants from preclinical models to human therapeutic applications (189).

#### 9.2.2 SKQ1

SkQ1 belongs to the class of mitochondriotropic antioxidants and shares a similar targeting rationale with MitoQ. While not as extensively researched as its predecessor, SkQ1 has shown significant efficacy in murine models and is currently undergoing clinical evaluation for the treatment of dry eye syndrome and macular degeneration (190).

In OXYS rats, dietary supplementation with SkQ1 has been demonstrated to inhibit the progression of retinopathy, a late-onset neurodegenerative condition of the retina that exhibits many clinical and pathological features of Alzheimer's disease (AD). These shared features include the accumulation of protein aggregates and the disruption of proteostasis (191). Furthermore, SkQ1 has been shown to enhance mitochondrial function and reduce oxidative stress in the retina, which are crucial factors in the pathogenesis of age-related macular degeneration (AMD) and other retinal degenerative diseases (192). This dual mechanism of action underscores the potential of SkQ1 as a promising therapeutic agent for retinal disorders associated with aging and oxidative stress.

Beyond Alzheimer's disease, the mitochondrial antioxidant SkQ1 is displaying therapeutic value in related degenerative disorders, offering a proof-of-concept for the TPP^+^ targeting platform. While SkQ1 has demonstrated efficacy in preclinical models for retinopathy and oligodendrocyte depletion (193, 194), it is currently in Phase II and III trials for ocular disorders such as macular degeneration. These trials are crucial for proving the long-term human safety of mitochondria-targeted antioxidants; nevertheless, the capacity of SkQ1 to achieve adequate blood-brain barrier (BBB) penetration to alter AD-specific neurodegeneration has to be clinically confirmed.

#### 9.2.3 SS-31 (elamipretide)

Elamipretide, also referred to as SS-31, MTP-131, or Bendavia, is a mitochondriotropic tetrapeptide developed to target mitochondrial dysfunction by binding to cardiolipin (CL) in the inner mitochondrial membrane (195). In the context of Alzheimer's disease, the buildup of amyloid-beta (Aβ) in cerebral mitochondria disrupts the electron transport chain (ETC), resulting in significant ATP depletion and bioenergetic impairment (196).

In laboratory studies conducted with N2a cells engineered to express mutant Aβ PP cDNA, elamipretide has been shown to promote the generation of new mitochondria and shield against the aggregation triggered by Aβ. These results align with earlier animal studies indicating that elamipretide could counteract the harmful effects of Aβ in the brain of individuals with Alzheimer's disease by fortifying the integrity of mitochondrial membranes and reviving respiratory activity (196) (Table 4).

**Table 4:** Mitochondrial structural and functional defects in Alzheimer's disease.

**Mitochondrial defect**	**Molecular driver**	**Functional outcome**	**Neuropathological effect**	**References**
ETC complex I dysfunction	Amyloid-β interaction	Reduced electron flow	Energy failure	(276)
ETC complex III damage	Oxidative stress	ROS amplification	Neuronal injury	(285)
ETC complex IV inhibition	Tau pathology	Reduced oxygen utilization	Synaptic loss	(301)
mtDNA mutations	Chronic ROS	Impaired transcription	Accelerated degeneration	(301)
Reduced membrane potential	Lipid peroxidation	ATP synthesis failure	Apoptosis	(302)
Excessive mitochondrial fission	Drp1 overactivation	Fragmented mitochondria	Synaptic vulnerability	(11)
Impaired fusion	Mfn1/2 downregulation	Loss of network integrity	Neuronal stress	(279)
Defective mitophagy	PINK1–Parkin failure	Damaged mitochondria accumulation	Cell death	(284)
Altered cristae structure	Cardiolipin loss	ETC inefficiency	Bioenergetic decline	(293)
Calcium buffering failure	MCU dysregulation	Excitotoxicity	Neuronal loss	(305)
Impaired axonal transport	Tau hyperphosphorylation	Energy deprivation at synapse	Cognitive deficits	(287)
Increased permeability transition	mPTP opening	Apoptosis initiation	Neurodegeneration	(303)
Reduced mitochondrial biogenesis	PGC-1α suppression	Low mitochondrial mass	Aging phenotype	(305)
Altered dynamics proteins	OPA1 loss	Structural instability	Network disruption	(296)
Reduced antioxidant enzymes	SOD2 decline	Oxidative damage	Disease acceleration	(303)
Impaired fatty acid oxidation	Enzyme downregulation	Lipid accumulation	Inflammation	(293)
Mitochondrial uncoupling	Proton leak	Heat generation	ATP depletion	(279)

While elamipretide (SS-31) substantially decreases mitochondrial ROS and maintains membrane integrity, its therapeutic efficacy appears to be limited in the wider neurovascular unit. The evidence suggests that SS-31 does not directly influence vascular remodeling, matrix metalloproteinase (MMP) activation, or BBB permeability—key drivers in the pathophysiology of cerebral microvascular hemorrhage (CMH) (197, 198). These findings highlight the complexities of CMH development and indicate that mitochondrial-targeted treatments alone may not be sufficient for total protection.

### 9.3 Enhancers of mitochondrial biogenesis

#### 9.3.1 PGCA-1

Mitochondrial biogenesis is crucial for controlling mitochondrial quantity, cellular turnover, and energy balance. PGC-1α acts as a key regulator in this process, playing a vital role in maintaining energy equilibrium and metabolic regulation. Its function is primarily controlled by Sirtuin 1 (SIRT1) and AMP-activated protein kinase (AMPK) (199). AMPK, serving as a sensor of the AMP/ATP ratio, phosphorylates PGC-1α during low-energy conditions, leading to the upregulation of glucose transport, fatty acid oxidation, and mitochondrial biogenesis (200, 201). Additionally, TFAM (Mitochondrial Transcription Factor A) plays a role in regulating mtDNA transcription and replication as part of the mitochondrial base excision repair process (202).

In the hippocampus of individuals with Alzheimer's disease (AD), the expression of PGC-1α, NRF1, NRF2, and TFAM is significantly decreased, indicating impaired mitochondrial biogenesis (203). In the context of AD development, the activity of PGC-1α is inversely related to Aβ levels, implying that its deficiency may contribute to amyloid accumulation (204).

#### 9.3.2 Exercise mimetics

Exercise mimetics are a type of pharmaceutical substances that aim to reproduce the physiological and neurological advantages of physical activity. These compounds are designed to mimic the effects of exercise by targeting the molecular pathways that are responsible for the beneficial effects of physical exertion (205). For example, certain mimetics can activate signaling pathways like AMPK or PGC-1α, which are typically activated during exercise (206).

The exact ways in which exercise mimetics help prevent Alzheimer's disease (AD) are still being studied, but several theories have been suggested. These include reducing neuroinflammation and oxidative stress, promoting the growth of new neurons and strengthening synaptic connections, and increasing the expression of neurotrophic factors. Additionally, it is believed that these compounds can decrease the buildup of Aβ plaques and neurofibrillary tangles, which are characteristic features of AD pathology (205, 207).

#### 9.3.3 NAD^+^ boosters

The hypothesis that metabolic disruption contributes to Alzheimer's disease (AD) is supported by the high metabolic demands of neurons and the age-related decline in NAD^+^ levels (208). Research in animal models indicates that NAD^+^ metabolism is severely compromised during AD progression. A systematic review of studies involving rodent models confirmed that treatment with NAD^+^ precursors successfully restores cerebral NAD^+^ concentrations, leading to marked improvements in learning and memory (209). These therapeutic effects are mediated by multiple mechanisms, including enhanced mitochondrial activity and the attenuation of oxidative stress, neuroinflammation, and apoptosis. The administration of the NAD^+^ precursor nicotinamide riboside (NR) to APP/PS1-mutant mice has been demonstrated to improve cognitive performance (209). Additionally, in Alzheimer's disease models where neuronal dysfunction was induced by intracerebroventricular injection of Aβ 1–42, treatment with nicotinamide (NAM) effectively prevented memory deficits (210).

There is a clear discrepancy between preclinical success and clinical proof for NAD^+^ precursors, according to a recent systematic review (211) of the 2018–2023 literature. Although nicotinamide riboside (NR) and nicotinamide mononucleotide (NMN) regularly reduce amyloid pathology and improve cognitive performance in murine models through Sirtuin-mediated mitochondrial biogenesis, their clinical validation is still in its early stages. Notably, the analysis found that there are currently no published human clinical studies using NMN in AD populations. On the other hand, successful modulation of neuroimaging biomarkers and plasma metabolic profiles has been shown in upcoming NR trials; nevertheless, they have not yet resulted in statistically meaningful, disease-modifying cognitive outcomes. These results highlight the fact that, although NAD^+^ restoration is a biologically valid approach, its clinical effectiveness is now only supported by surrogate biomarkers rather than cognitive recovery.

### 9.4 Ketogenic strategies and alternative fuel provision

#### 9.4.1 Keto diet

Oxidative stress and mitochondrial dysfunction are key factors in neurodegeneration, leading to increased production of reactive oxygen species (ROS) that damage cellular components such as proteins, lipids, and nucleic acids (212). The ketogenic diet (KD) may have neuroprotective effects by reducing glycolysis and increasing the production of ketone bodies (KBs). Metabolic ketosis can improve mitochondrial respiration, reduce ROS production, and prevent dysfunction of Complex I.

The effectiveness of the KD was assessed in two transgenic mouse models: Tg4510 mice (tauopathy model) and APP/PS1 mice (amyloidosis model). After 3 months of dietary intervention, behavioral tests showed that mice on the KD performed significantly better on the rotarod compared to control groups, regardless of their specific genotype (213). Despite its therapeutic potential, critics of the ketogenic diet (KD) raise concerns about the high intake of saturated fats and processed meats, which are traditionally associated with increased risks of cardiovascular disease, diabetes, and Alzheimer's disease (AD) (214). However, recent research suggests that a well-formulated KD may not have a negative impact on metabolic health. Clinical data show that participants following a KD can achieve significant reductions in body weight and HbA1c levels, as well as increases in HDL cholesterol (215). These conflicting findings may be due to differences in the fatty acid composition of various ketogenic protocols.

#### 9.4.2 Exogenous ketone esters

Glucose hypometabolism, which refers to the impaired utilization of glucose in the brain, is a characteristic feature of Alzheimer's disease (216). When this metabolic deficiency occurs, the brain can use ketone bodies as an alternative source of fuel to meet its energy needs. In particular, the ketone body β-hydroxybutyrate has been found to reduce the generation of reactive oxygen species (ROS) by regulating mitochondrial Complex I. Additionally, the metabolism of ketone bodies increases the expression of uncoupling proteins (UCPs), which improves the efficiency of the electron transport chain during oxidative phosphorylation.

#### 9.4.3 Medium chain triglycerides

Medium-chain triglycerides (MCTs) are a powerful source for producing ketone bodies. The enzyme lipase breaks down MCTs into medium-chain fatty acids (MCFAs) (217). Unlike long-chain fatty acids, MCFAs are absorbed and metabolized more effectively as they do not depend on the carnitine palmitoyltransferase transport system for entering mitochondria (218). MCTs can be introduced through dietary adjustments or external supplementation. The increasing prevalence of Alzheimer's disease (AD) has created a pressing demand for safe and efficient therapeutic treatments. Several clinical trials have explored the potential of Medium Chain Triglycerides (MCTs) in enhancing cognitive function in AD and Mild Cognitive Impairment (MCI) patients, but the findings are still a topic of discussion.

Two systematic reviews have been conducted to consolidate the overall impact of MCTs on cognition. A comprehensive review of literature up to March 2019 suggests that MCT supplementation may improve cognitive clarity and performance in individuals with AD and MCI (219).

### 9.5 Modulators of mitophagy

#### 9.5.1 Urolithin A

Urolithin A (UA) is a natural metabolite produced by the gut microbiota through the conversion of ellagitannins and ellagic acid, polyphenolic compounds abundant in pomegranates, berries, and nuts (220). Therapy with UA has been shown to increase mitophagy in the brains of Alzheimer's disease (AD) murine models, such as APP/PS1 and 3xTgAD mice, as well as in nematode models. These cellular improvements are associated with significant enhancements in learning and memory (35). Due to its strong antioxidant properties (221) and its role as a mitophagy inducer (222), UA exhibits anti-aging effects, enhances mitochondrial activity, and improves muscular performance.

**Figure 5:**
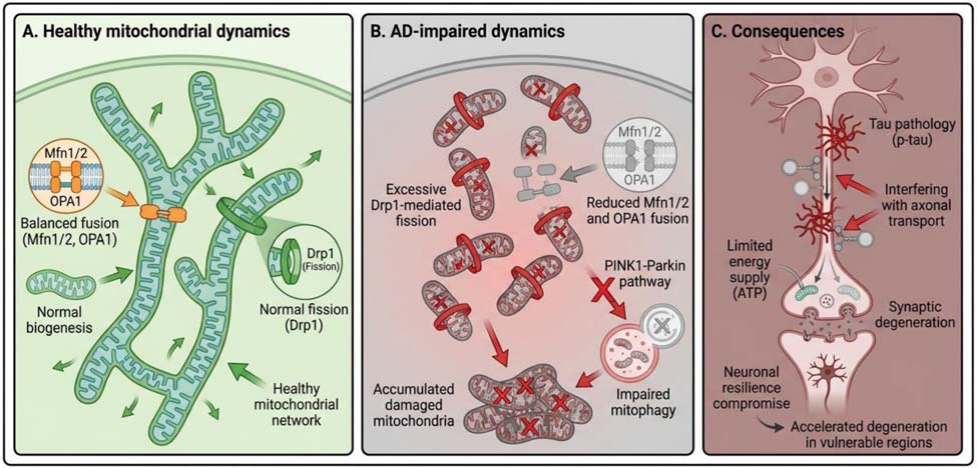
Impaired mitochondrial dynamics, mitophagy, and quality control.

Importantly, UA has the ability to penetrate the blood-brain barrier (BBB) and has been recognized as a potent inducer of both autophagy and mitophagy in the central nervous system (223). Additionally, UA has demonstrated neuroprotective effects against oxidative stress caused by hydrogen peroxide (H2O2), thereby safeguarding neuronal survival in the face of elevated levels of reactive oxygen species (ROS) (Figure 5).

According to recent research (224), urolithin A (UA) repairs the lysosomal-mitochondrial axis, which promotes neuroprotection. UA has been demonstrated to restore long-term potentiation (LTP) and lower Aβ plaque burden in AD animal models by normalizing lysosomal acidity and modulating cathepsin Z (Ctsz) expression. The translation of UA from murine models to human clinical efficacy is still the final obstacle to its approval as a potential AD treatment, even if these findings provide a strong molecular basis.

#### 9.5.2 Spermidine

Spermidine is an essential polyamine derived from putrescine and acts as a precursor to spermine (225). Research suggests that this polyamine possesses significant lifespan-extending, neuroprotective, and cardioprotective properties (226).

The main mechanism responsible for the longevity-promoting effects of spermidine is the stimulation of autophagy (227). In addition to inducing autophagic flux, spermidine reduces oxidative stress-related damage in aging models by inhibiting excessive reactive oxygen species (ROS) production and preventing necrotic cell death (227). Moreover, spermidine has been found to mitigate brain aging by regulating mitochondrial structure and enhancing respiratory function under cellular stress conditions (228). In the APP/PS1 mouse model of Alzheimer's disease-related amyloidopathy, long-term dietary supplementation with spermidine has been shown to decrease neuroinflammation and significantly decrease the levels of toxic, soluble amyloid-beta (Aβ) (229).

The results of the 12-month phase 2b randomized controlled study (230) showed that oral spermidine supplementation did not significantly improve mnemonic discrimination or regulate AD biomarkers in older persons with subjective cognitive decline (SCD), despite strong preclinical performance. The major cognitive objectives were not reached, despite the fact that the intervention was safe and exploratory analyses indicated possible minor advantages for verbal memory and inflammation. These results highlight a substantial translational gap, indicating that the metabolic deterioration linked to early-stage AD may not be reversible with existing dosages or intervention schedules.

#### 9.5.3 Rapalogs and calorie restriction mimetics

Rapamycin (sirolimus), a macrolide compound derived from Streptomyces hygroscopicus, is a potent inhibitor of the mechanistic target of rapamycin (mTOR) complex. Rapamycin has demonstrated significant neuroprotective effects by enhancing autophagic flux, which aids in clearing proteotoxic aggregates associated with various neurodegenerative disorders. In addition to its role in aggregate clearance, rapamycin's neuroprotective properties are attributed to its ability to reduce neuroinflammation linked to proteinopathy. Studies in murine models of mitochondrial Leigh syndrome (231), cerebral palsy (232), and tauopathy (233) have shown that rapamycin treatment can decrease microglial activation and improve clinical symptoms.

Rapamycin's effectiveness in AD seems to be controlled by a narrow therapeutic window; data suggests that in late disease, its advantages may disappear or even turn harmful (234). Clinical attention has switched to prevention in MCI cohorts or middle-aged individuals displaying early MAPT (tau) tangles and amyloid deposition since mTOR inhibition is more efficient at removing early-stage aggregates (235). Rapamycin's safety in MCI is currently being evaluated in a Phase I trial (NCT04200911), but the “translational hurdle” determining whether the medication can continue to engage its targets once neurodegeneration reaches a late-stage plateau—remains (236, 237). Ultimately, rather than acting as a reversal drug for dementia that has already developed, rapamycin probably acts as a delay mechanism for early AD.

Caloric restriction (CR) also demonstrates strong anti-inflammatory effects in the central nervous system. In studies involving cortical injury and lipopolysaccharide (LPS)-induced neuroinflammation, CR has been shown to significantly reduce microglial activation (238). In models of Alzheimer's disease (AD), CR offers multiple benefits: it increases the production of antioxidants and anti-apoptotic proteins, boosts histone deacetylase activity, and elevates levels of brain-derived neurotrophic factor (BDNF). Moreover, CR influences insulin signaling pathways, enhances DNA repair mechanisms, and inhibits the mTOR pathway, collectively leading to a decrease in Aβ plaque accumulation and a reduction in tau phosphorylation. These systemic and cellular changes effectively counteract Aβ-induced cytotoxicity and support long-term neuronal survival (239).

### 9.6 Lifestyle interventions

#### 9.6.1 Aerobic exercises

Aerobic exercise has been found to have a significant positive impact on memory and executive function in individuals with Mild Cognitive Impairment (MCI) (240). However, the extent of cognitive improvement in older adults with Alzheimer's disease (AD) dementia is still under investigation, with reported effects varying from minimal (241) to significant (242). In addition to cognitive benefits, aerobic exercise also affects non-hallmark AD pathologies by reducing oxidative stress, suppressing neuroinflammation, and improving glucose hypometabolism (243).

Despite the strong molecular foundations, findings from randomized controlled trials (RCTs) on cognitive effectiveness in Alzheimer's disease (AD) have shown inconsistency (244). Meta-analyses of these RCTs indicate that aerobic exercise offers moderate cognitive advantages (245). Additionally, recent research suggests a potential correlation between the intensity of aerobic exercise and cognitive maintenance in individuals with mild cognitive impairment (MCI) and AD dementia (246).

#### 9.6.2 Time-restricted eating (TRE)

Time-restricted eating (TRE) is a type of intermittent fasting (IF) that has garnered considerable attention for its emphasis on meal timing rather than calorie counting. TRE involves restricting the daily eating window to 8–12 h, followed by a fasting period of 12–16 h within a 24-h cycle, during which only water or non-caloric beverages are consumed (247). In Alzheimer's disease (AD) mouse models, TRE has been found to increase levels of β-hydroxybutyrate (BHB), reduce blood glucose levels, and enhance sleep quality compared to control groups (248). Additionally, in mice with APP mutations, the implementation of TRE alongside a 30–40% reduction in calorie intake significantly mitigated the accumulation of Aβ plaques (249) (Table 5).

**Table 5:** Emerging interventions targeting metabolic and mitochondrial dysfunction in Alzheimer's disease.

**Intervention strategy**	**Target pathway**	**Mechanism of action**	**Therapeutic potential**	**References**
Intranasal insulin	Insulin signaling	Enhances neuronal glucose uptake	Cognitive improvement	(305)
Metformin	AMPK activation	Improves insulin sensitivity	Disease modification	(296)
Ketogenic diet	Alternative fuel	Provides ketone bodies	Energy restoration	(284)
Mitochondria-targeted antioxidants	ROS reduction	Protects ETC function	Neuroprotection	(294)
NAD^+^ precursors	Redox balance	Enhances mitochondrial repair	Anti-aging effect	(287)
PGC-1α activators	Biogenesis	Increases mitochondrial mass	Functional recovery	(301)
Exercise	Metabolic flexibility	Enhances insulin sensitivity	Slows decline	(293)
Caloric restriction	AMPK–SIRT1 axis	Improves efficiency	Longevity benefit	(287)
Coenzyme Q10	ETC support	Improves electron transport	ATP production	(302)
Omega-3 fatty acids	Membrane stability	Reduces inflammation	Synaptic protection	(284)
Mitophagy enhancers	PINK1–Parkin	Removes damaged mitochondria	Cellular resilience	(284)
GLP-1 agonists	Glucose regulation	Neuroprotective signaling	Clinical trials	(294)
Lactate supplementation	Energy shuttle	Supports neuronal metabolism	Synaptic function	(296)
Anti-inflammatory diets	Metabolic inflammation	Reduces cytokines	Disease modulation	(302)
SIRT1 activators	Mitochondrial repair	Enhances stress resistance	Cognitive preservation	(293)
Gene therapy	Mitochondrial genes	Restores function	Experimental promise	(301)
Combination therapies	Multi-target approach	Addresses complex pathology	Translational potential	(284)

#### 9.6.3 Anti inflammatory diets

Compelling evidence suggests that strict adherence to the Mediterranean diet (MedDiet) is linked to reduced cognitive decline and a lower incidence of Alzheimer's disease (AD) in the elderly (250). In a prospective study involving 3,831 elderly participants, greater compliance with the MedDiet and Dietary Approaches to Stop Hypertension (DASH) protocols, particularly through high consumption of fruits, vegetables, nuts, legumes, and whole grains, was significantly associated with higher Mini-Mental State Examination (MMSE) scores. Moreover, findings from comprehensive systematic reviews and meta-analyses indicate that following the MedDiet rigorously is associated with a decreased risk of mild cognitive impairment (MCI) and AD, as well as a lower likelihood of progression from MCI to clinical AD (251). Similarly, the MIND diet, which combines elements of the MedDiet and DASH and emphasizes neuroprotective foods like berries and green leafy vegetables, is linked to a reduced incidence of AD and a slower rate of cognitive decline (252).

Cross-sectional research suggests that a lower ratio of omega-6 to omega-3 fatty acids can influence the inflammatory response, leading to improved hippocampus-dependent spatial memory and faster learning in virtual navigation tasks (253). Diets like the Mediterranean Diet (MedDiet), which have a low omega-6 to omega-3 ratio, have been found to reduce the risk of Alzheimer's disease (AD) and slow down cognitive decline (253).

### 9.7 Limitations of dietary interventions

Current evidence supports the hypothesis that ketogenic diets (KDs) can enhance cognitive outcomes in patients with Alzheimer's disease (AD). However, the effectiveness of this therapeutic approach is influenced by the stage of AD, disease progression, and the presence of the ApoE4 genotype, which significantly affects the metabolic response to dietary interventions. Despite maintaining adequate caloric intake, the strict carbohydrate restriction necessary for achieving ketosis may compromise the overall quality of the diet. Low-carbohydrate regimens often lack essential micronutrients such as thiamine, folate, vitamins A, E, B6, and K, as well as minerals like calcium, magnesium, and iron. Additionally, KDs are typically low in dietary fiber.

Long-term adherence to very-low-carbohydrate diets has been associated with increased risks of all-cause mortality, cardiovascular events, and cancer-related mortality (254). As a result, these diets are not recommended for certain patient populations, including those with a history of hypertriglyceridemia-related acute pancreatitis or severe hypertriglyceridemia. Caution is also advised for patients taking sodium-glucose cotransporter 2 (SGLT2) inhibitors or diuretics, as they may be at higher risk of metabolic complications and electrolyte imbalances.

### 9.8 Drugs and blood brain barrier efficiency issues

The physiological properties of the blood-brain barrier (BBB) undergo significant changes during the progression of Alzheimer's disease (AD) (Table 6). It is crucial to use various cellular models to study these disease-specific states, as BBB permeability and drug transport kinetics can differ based on the underlying pathology.

**Table 6:** Clinical and neuroimaging evidence of metabolic dysfunction in Alzheimer's disease.

**Evidence type**	**Measurement tool**	**Key observation**	**Clinical relevance**	**References**
FDG-PET imaging	Glucose uptake	Regional hypometabolism	Early diagnosis	(287)
MRI spectroscopy	Metabolite ratios	Reduced NAA	Neuronal loss	
CSF insulin levels	Immunoassay	Altered insulin signaling	Metabolic impairment	(296)
Plasma glucose	Blood analysis	Dysglycemia	Risk stratification	(293)
HOMA-IR	Insulin resistance index	Elevated values	AD risk	(301)
Brain lactate levels	MRS	Accumulation	Mitochondrial stress	(284)
ATP levels	Spectrophotometry	Reduced synthesis	Energy failure	(287)
Cognitive testing	MMSE, MoCA	Decline correlates with hypometabolism	Disease severity	(305)
Amyloid PET	Plaque deposition	Metabolism–amyloid link	Pathological confirmation	(294)
Tau PET	Neurofibrillary tangles	Metabolic disruption	Disease staging	(302)
CSF lactate/pyruvate ratio	Metabolic assay	Redox imbalance	Mitochondrial dysfunction	(293)
Functional MRI	Network connectivity	Reduced efficiency	Cognitive impairment	(301)
EEG metabolic markers	Neural oscillations	Reduced synchronization	Synaptic failure	(294)
Longitudinal PET	Metabolic decline rate	Disease progression	Prognosis	(305)
APOE genotype	Genetic testing	Altered lipid metabolism	Risk modifier	(284)
Peripheral insulin sensitivity	Clamp studies	Reduced sensitivity	Brain–body link	(296)
Clinical trials	Metabolic endpoints	Modest cognitive benefit	Therapeutic relevance	(302)

In the future, Drug Delivery Systems (DDS) will be increasingly important for targeting the brain, as they can overcome traditional delivery barriers. Currently, multi-functionalized DDS, which incorporate multiple targeting ligands or responsive elements, are more effective than single-functionalized ones. Additionally, combining different DDS platforms shows promise for improving therapeutic outcomes. An example is the drug-in-cyclodextrin-in-liposome system, which combines the stabilization properties of cyclodextrins with the biocompatibility and transport abilities of liposomes to achieve better therapeutic results (255).

### 9.9 Limitations of clinical implementations

Preclinical testing of multi-target therapies poses additional challenges, as current animal models and testing protocols often do not accurately capture the complex interactions of multiple targets (256). Furthermore, the stringent regulatory requirements for new multi-target therapies make the approval process more suitable for single-target drugs, necessitating adjustments that could hinder progress and increase costs. The primary obstacle to widespread adoption of this approach is the substantial research and development expenses, along with the potential high costs involved in bringing it to market (257).

## 10 Integrating clinical and mechanistic insights

Positron emission tomography (PET) and magnetic resonance spectroscopy (MRS) are key neuroimaging techniques used to study cerebral characteristics that indirectly reflect mitochondrial activity (258). Among these, the PET tracer [18F] 2-fluoro-2-deoxy-d-glucose (FDG) is commonly used in Alzheimer's disease (AD) diagnosis. FDG allows for a comprehensive assessment of whole-brain glucose utilization, although it is important to recognize that FDG-PET signals do not specifically target mitochondrial integrity. In individuals with AD, there is a strong association between cerebral blood flow and glucose metabolism (259). Consequently, vascular dysfunction, including endothelial and blood-brain barrier (BBB) impairments, can lead to reduced blood flow, depriving neurons of the necessary substrates for ATP production (260). Clinical FDG-PET results often show significant glucose hypometabolism in the inferior parietal cortex and posterior cingulate cortex.

Over 210 rodent models have been developed to mimic the clinical features of Alzheimer's disease (AD), but their success in translating to human treatments is limited. Despite efforts to understand the molecular mechanisms of the disease in these models, potential treatments that show promise in rodents often fail in human clinical trials (261). Bioenergetic failure and impaired glucose metabolism are early signs of AD. While it may seem logical to consider glucose supplementation as a strategy, impaired cerebral glucose metabolism in AD is often linked to systemic hyperglycemia, as seen in diabetes, rather than hypoglycemia. Therefore, focusing solely on glucose levels is not a viable treatment approach. Instead, targeting the restoration of compromised neuronal bioenergetics and enhancing mitochondrial energy metabolism offer more promising therapeutic options.

## 11 Future prospects

A formal framework has recently been proposed for the application of precision medicine (PM) to neurodegenerative diseases (262). This approach moves away from the traditional “one-size-fits-all” model and instead focuses on personalized, biomarker-guided targeted therapies and best practices tailored to an individual's unique biological profile (263). Due to the complex pathophysiology of Alzheimer's disease (AD), finding a single universal therapeutic agent is increasingly seen as unlikely, a realization already acknowledged in oncology and cardiology. To effectively implement PM, it is crucial to take an interdisciplinary systems biology (SB) perspective, supported by systems neurobiology, to map the intricate networks involved in neurodegeneration (264).

### 11.1 Precision medicine and multi-omics integration in Alzheimer's disease

While multi-omic investigations have transformed oncology over the past decade, their application in AD is still in its early stages (265). Despite the emergence of several human cohort studies in the last 5 years, few are truly integrative. Many studies focus on proteomic, transcriptomic, or lipidomic changes in isolation rather than integrating them into a comprehensive biological model. For example, research utilizing microglial extracellular vesicles (EVs) from cryopreserved AD brain tissue analyzed lipidomic, transcriptomic, and proteomic datasets to illustrate how microglial activity shifts in late-stage AD, impacting the clearance of tau-laden neuronal debris (266). This suggests that EVs could serve as a source of multi-omic data and a potential diagnostic indicator of disease progression.

Blood (plasma/serum) remains the most practical biological matrix for large-scale exploratory investigations. Blood-based biomarkers offer significant advantages over neuroimaging or cerebrospinal fluid (CSF) analysis, particularly in terms of cost-effectiveness and accessibility. As a result, they are being positioned as the initial step in a multi-stage diagnostic approach, similar to established protocols in cardiovascular and infectious diseases (267). To successfully integrate these markers into clinical practice, the primary focus should be on establishing Contexts of Use (COUs)—formal statements that define the specific purpose and manner of biomarker application in drug development and clinical diagnosis (268).

Multi-omic studies of AD are still in their early stages, despite the fact that multi-omics has been utilized for more than 10 years to examine changes in other diseases, particularly in the field of oncology (265). However, a number of multi-omics research using human cohorts and data in the context of AD have been conducted over the last 5 years. However, few of these research are genuinely integrative. Despite taking into account several biological levels, they only looked at these changes separately. For example, a study (266) analyzed proteomics, transcriptomics and lipidomics data derived from microglial extracellular vesicles acquired from cryopreserved brain tissue with neuropathologically diagnosed AD. They demonstrated how neuronal debris, including tau in extracellular vesicles, is released when microglial activity is altered in the latter stages of AD. This implies that extracellular vesicles may serve as a possible readout of disease state in addition to being a source of future omics data.

However, a real integrative strategy has been the focus of some recent investigations. Crucially, researchers (269) have analyzed transcriptomics and genomes from AD patients' brain tissue using this method. They have demonstrated that single-omics techniques fail to identify the majority of pathways implicated in AD pathogenesis. In fact, it is more probable to find important biological hubs or pathway changes that are pertinent to disease when several weak signals from several individual molecules are combined. For instance, ATP6V1A, which encodes an ATPase component, was identified as a crucial network regulator implicated in several protein networks linked to AD in a study (304) using post-mortem brain tissue samples with confirmed AD. This shows that this gene is not only a possible risk factor for AD but could also serve as a target for disease alterations, emphasizing the efficacy of integrative techniques for uncovering potential disease modifying medication targets. Furthermore, by finding 20 SNPs linked to amyloid deposition and frontal brain shrinkage, the gap between genomics and clinical imaging was successfully closed (270). When taken as a whole, these studies demonstrate the existence of repeatable metabolic and proteomic signatures; nonetheless, the lack of standardized techniques and the requirement for validation in larger, more varied patient cohorts continue to impede their therapeutic application. In the end, overcoming major obstacles in data standardization, validating findings across various longitudinal cohorts, and creating easily accessible, reasonably priced diagnostic tools are necessary to close the gap between multi-omic discovery and clinical practice.

## 12 Conclusion

Alzheimer's disease is a multifaceted condition characterized by the accumulation of abnormal proteins, neuronal loss, and persistent inflammation. It impacts not only the brain but also the entire body due to oxidative stress and metabolic disruptions. Memory and cognitive decline primarily stem from the impairment of brain regions responsible for learning and synaptic transmission. Mitochondrial dysfunction is a key factor in these alterations, underscoring the significance of mitochondria as a valuable therapeutic target. Interventions aimed at preserving or enhancing mitochondrial function could mitigate neuronal harm and bolster cognitive well-being, presenting a hopeful avenue for future Alzheimer's disease management.
